# Scalable whole-exome sequencing of cell-free DNA reveals high concordance with metastatic tumors

**DOI:** 10.1038/s41467-017-00965-y

**Published:** 2017-11-06

**Authors:** Viktor A. Adalsteinsson, Gavin Ha, Samuel S. Freeman, Atish D. Choudhury, Daniel G. Stover, Heather A. Parsons, Gregory Gydush, Sarah C. Reed, Denisse Rotem, Justin Rhoades, Denis Loginov, Dimitri Livitz, Daniel Rosebrock, Ignaty Leshchiner, Jaegil Kim, Chip Stewart, Mara Rosenberg, Joshua M. Francis, Cheng-Zhong Zhang, Ofir Cohen, Coyin Oh, Huiming Ding, Paz Polak, Max Lloyd, Sairah Mahmud, Karla Helvie, Margaret S. Merrill, Rebecca A. Santiago, Edward P. O’Connor, Seong H. Jeong, Rachel Leeson, Rachel M. Barry, Joseph F. Kramkowski, Zhenwei Zhang, Laura Polacek, Jens G. Lohr, Molly Schleicher, Emily Lipscomb, Andrea Saltzman, Nelly M. Oliver, Lori Marini, Adrienne G. Waks, Lauren C. Harshman, Sara M. Tolaney, Eliezer M. Van Allen, Eric P. Winer, Nancy U. Lin, Mari Nakabayashi, Mary-Ellen Taplin, Cory M. Johannessen, Levi A. Garraway, Todd R. Golub, Jesse S. Boehm, Nikhil Wagle, Gad Getz, J. Christopher Love, Matthew Meyerson

**Affiliations:** 1 Eli and Edythe L. Broad Institute of MIT and Harvard, 415 Main Street, Cambridge, 02142 MA USA; 2 Koch Institute for Integrative Cancer Research, Massachusetts Institute of Technology, 500 Main Street, Cambridge, 02142 MA USA; 3 Dana-Farber Cancer Institute, 450 Brookline Avenue, Boston, 02215 MA USA; 4 Harvard Medical School, 250 Longwood Avenue, Boston, 02115 MA USA; 5 Massachusetts General Hospital, 55 Fruit Street, Boston, 02129 MA USA; 6 Brigham and Women’s Hospital, 75 Francis Street, Boston, 02115 MA USA; 7 Howard Hughes Medical Institute, 4000 Jones Bridge Road, Chevy Chase, 20815 MD USA

## Abstract

Whole-exome sequencing of cell-free DNA (cfDNA) could enable comprehensive profiling of tumors from blood but the genome-wide concordance between cfDNA and tumor biopsies is uncertain. Here we report ichorCNA, software that quantifies tumor content in cfDNA from 0.1× coverage whole-genome sequencing data without prior knowledge of tumor mutations. We apply ichorCNA to 1439 blood samples from 520 patients with metastatic prostate or breast cancers. In the earliest tested sample for each patient, 34% of patients have ≥10% tumor-derived cfDNA, sufficient for standard coverage whole-exome sequencing. Using whole-exome sequencing, we validate the concordance of clonal somatic mutations (88%), copy number alterations (80%), mutational signatures, and neoantigens between cfDNA and matched tumor biopsies from 41 patients with ≥10% cfDNA tumor content. In summary, we provide methods to identify patients eligible for comprehensive cfDNA profiling, revealing its applicability to many patients, and demonstrate high concordance of cfDNA and metastatic tumor whole-exome sequencing.

## Abstract (web summary)

Identifying the mutational landscape of tumours from cell-free DNA in the blood could help diagnostics in cancer. Here, the authors present ichorCNA, software that quantifies tumour content in cell free DNA, and they demonstrate that cell-free DNA whole-exome sequencing is concordant with metastatic tumour whole-exome sequencing.

## Introduction

To enable precision medicine, it must be possible to routinely sample and sequence patients’ tumors. A major challenge, however, is that repeated tumor biopsies are often intractable, particularly for patients with metastatic cancer. Significant progress has been made for tracking previously identified tumor mutations in cell-free DNA (cfDNA)^1–7^, but whether cfDNA can capture the genetic diversity of cancer has not been systematically explored. Whole-exome sequencing (WES) of cfDNA has demonstrated potential to detect clinically relevant alterations^8–10^, but its broader application has been challenging because the yield and fraction of tumor-derived cfDNA (“tumor fraction”) vary substantially. Furthermore, genome-wide comparisons of cfDNA and tumor biopsies are limited in both quantity and comprehensiveness. For these reasons, it remains unknown to what degree WES of cfDNA would be applicable to patients with metastatic cancer and whether WES of cfDNA may complement or replace WES of a surgical tumor biopsy.

Previous benchmarking has shown that somatic alterations can be detected with reasonable sensitivity using standard depths of WES (~150× coverage) from tumor samples harboring at least ~5–10% tumor content^11^. Given the variability in cfDNA tumor fractions, we reasoned that advanced screening for tumor content would be needed to make WES of cfDNA possible at scale. Many previous approaches to screening for cancer-derived cfDNA have focused on targeted detection of somatic single nucleotide variants (SSNVs) in recurrently mutated cancer genes^6, 7^. However, somatic copy number alterations (SCNAs) may be more generally applicable as the vast majority of metastatic cancers harbor arm-level somatic SCNAs^12^. Groups have demonstrated that it is feasible to detect SCNAs using 0.1× whole-genome sequencing of cfDNA^13–15^, but methods to estimate tumor fraction require ~100-fold greater coverage^16, 17^. We hypothesized that being able to estimate tumor fraction from 0.1× sequencing coverage (ultra-low-pass whole-genome sequencing, ULP-WGS) could enable cost-effective screening for the existence of a significant amount of tumor-derived cfDNA in a substantial fraction of patients with metastatic cancer and thus, calibrate the application of WES.

**Fig. 1:**
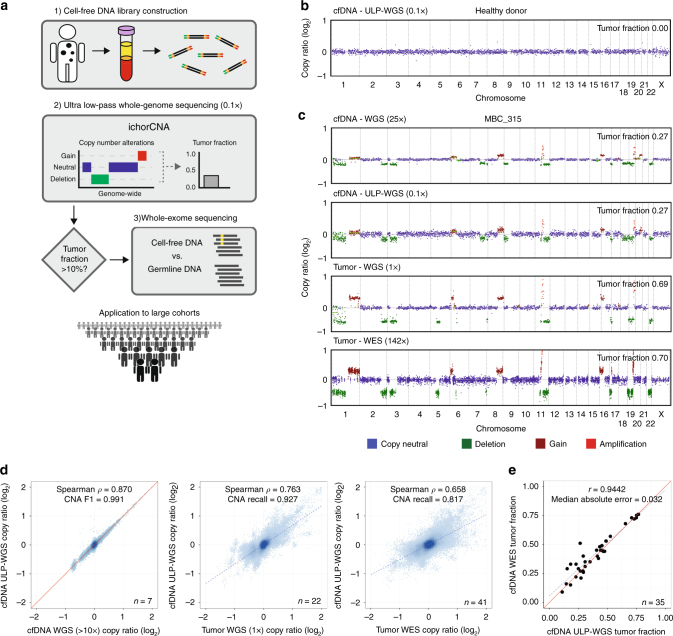
Copy number and tumor fractions from ULP-WGS. **a** cfDNA workflow. **b** Genome-wide copy number from 0.1× ULP-WGS of cfDNA from a healthy donor. **c** Genome-wide copy number from 25× WGS and 0.1× WGS of cell-free DNA from a metastatic breast cancer patient (MBC_315), and 1× WGS and WES of matched tumors from this patient. SCNA for tumor WES and cfDNA 25× coverage WGS were predicted using TITAN^17^ (“Methods”). **d** Comparison of copy ratios between ULP-WGS of cfDNA with deep (>10×) WGS of the same cfDNA sample, WGS (1×) of matched tumors from 22 metastatic breast cancer (MBC) patients, and WES (average mean target coverage 173×) of matched tumors from 41 MBC and prostate cancer (CRPC) patients. Log_2_ copy ratios were computed as normalized read coverage for each 1 Mb (WGS/ULP-WGS) and the mean of overlapping 50 kb bins (WES) after adjustment for tumor fraction/purity. The correlation of copy ratios between tumor and cfDNA was computed using Spearman rank correlation (coefficient *ρ*). *F*-measure (F1) is the harmonic mean of the CNA positive predictive value (precision) and sensitivity (recall) performance. Recall is defined as the proportion of SCNA gain/loss in tumor biopsy also observed in ULP-WGS of cfDNA (“Methods”). **e** Comparison of tumor fractions estimated from ULP-WGS and WES of cfDNA. Samples (*n* = 35) with similar tumor ploidy (difference < 0.75 and ploidy ≥1.5) estimated in both ULP-WGS and tumor WES are shown. The correlation between the two data types was calculated using Pearson correlation (coefficient *r*). Red line denotes *y* = *x*. WES tumor fractions were estimated using ABSOLUTE^16^ (shown) and TITAN (Supplementary Fig. 15, Supplementary Data 6)

Here we develop an analytical approach, ichorCNA, to quantify tumor fraction in cfDNA without prior knowledge of SSNVs or SCNAs in patients’ tumors from ULP-WGS (Fig. 1a, “Methods”). We apply ichorCNA to determine which cfDNA samples have sufficient tumor content (>10%) for WES. Subsequent analysis of WES of cfDNA and matched tumor biopsies from 41 patients demonstrates that cfDNA provides a suitable proxy for a tumor biopsy. Further examination of 1439 blood samples from 520 patients with metastatic breast or prostate cancer using ichorCNA reveals >30% of blood samples and >40% of patients to have sufficient tumor fraction for standard depths of WES of cfDNA.

## Results

### ichorCNA provides accurate measure of cfDNA tumor fraction

Our process begins with patient blood collection, separation of plasma from blood, extraction of cfDNA from plasma and germline DNA (gDNA) from blood, and construction of cfDNA libraries (Fig. 1a, “Methods”). We found that the size distribution (Supplementary Fig. 1) and yields of cfDNA from metastatic cancer patients (median = 7.01, range = 0.00–547.82 ng/mL plasma, *n* = 1684) and healthy donors (HD) (median = 2.34, range = 0.55–21.27 ng/mL plasma, *n* = 27) were consistent with previous reports^18, 19^ (Supplementary Data 1). We optimized our library construction protocol for 5 ng of cfDNA input; 92.2% of cancer patients and 77.8% of healthy donors had ≥5 ng of cfDNA per 4 mL of plasma. Only 1% of each cfDNA sequencing library was then used for ULP-WGS to screen for tumor content.

ichorCNA simultaneously predicts segments of SCNA and estimates of tumor fraction while accounting for subclonality and tumor ploidy (“Methods”). To evaluate the performance of ichorCNA, we used ULP-WGS of cfDNA (Fig. 1b, c) and whole-genome sequencing of cfDNA (10×–48×, *n* = 7) and matched tumor biopsies (1×, *n* = 22) from metastatic breast and prostate cancer patients and healthy donors as benchmark data sets (Fig. 1c, Supplementary Fig. 2). We found highly concordant megabase-scale copy number (sensitivity > 0.92, Fig. 1d, Supplementary Figs. 3–5), including identification of chromothripsis (Supplementary Fig. 6). Tumor fraction estimates from ULP-WGS of cfDNA were also concordant with WGS of the same sample (Supplementary Fig. 3).

To further evaluate how ULP-WGS of cfDNA compares with the metastatic tumor, we performed standard WES of matched tumor biopsies (average mean target coverage 173×) from 41 patients with metastatic breast and prostate cancers who had a cfDNA sample with ≥0.1 tumor fraction (Supplementary Data 2). The cfDNA of the 41 selected cases had a median tumor fraction of 30.8% as estimated by ichorCNA. We observed that the majority of large, megabase-scale SCNAs detected by ULP-WGS of cfDNA was present in the metastatic tumors (median sensitivity 0.82, Spearman *ρ* = 0.66, Fig. 1d, Supplementary Figs. 5 and 7).

Using in silico mixing of up to 50 cancer patient and 22 healthy donor cfDNA samples to generate 2400 mixtures across a series of benchmarking data sets (“Methods”), we demonstrated the accurate estimation of tumor fraction (median deviation from expected ≤ 0.014) and detection of SCNAs at 0.1× coverage (Supplementary Figs. 8–13, Supplementary Data 3). ichorCNA has a sensitivity of 0.91 (Clopper–Pearson 95% confidence interval [0.88–0.93]) for classifying the in silico mixing samples with a tumor fraction >0.10, and has a specificity of 1.00 [0.85–1.00] for predicting a tumor fraction of <0.10 in 22 healthy donor (Supplementary Figs. 12 and 13). We also determined a lower limit of 0.03 tumor fraction for detecting the presence of tumor by using arm-level (>100 Mb) events from as few as one copy gain plus one copy loss (Supplementary Fig. 14). When using this 0.03 tumor fraction estimate cut-off, ichorCNA achieves a sensitivity of 0.95 [0.94–0.96] for detecting presence of tumor and a specificity of 0.91 [0.71–0.99] for correctly classifying a healthy donor (Supplementary Figs. 12 and 13). Our results suggest that the application of ichorCNA to ULP-WGS of cfDNA offers an accurate approach to detect SCNAs that are reflective of tumor biopsies and provides accurate estimates of tumor fractions, potentially even in cancer types with few SCNAs.

### Tumor and cfDNA exomes exhibit high concordance

We next performed WES of cfDNA (average mean target coverage 191×) from the same 41 patients with matched metastatic breast and prostate tumor biopsies (Supplementary Data 2) and detected somatic alterations (SSNVs and SCNAs, Supplementary Data 4 and 5). First, we compared ULP-WGS and WES of cfDNA and found high concordance of tumor fraction estimates (Pearson’s *r* = 0.94, Fig. 1e, Supplementary Figs. 15 and 16, Supplementary Data 6) and predicted SCNAs (median *F*-measure = 0.95, Supplementary Fig. 5). Furthermore, the predicted number of alterations in cfDNA and metastatic biopsies for non-silent SSNVs (median 50 vs. 63) and the fraction of genome altered by SCNA (47% vs. 44%) were consistent (Wilcoxon rank-sum test *p* > 0.5, Supplementary Fig. 17), which are similar to previous reports for these tumor types^20, 21^. We also performed WES of cfDNA from 12 healthy donors (average mean target coverage 126×) and observed a low false positive rate of SSNVs (median 0.03 non-silent SNVs/Mb, “Methods”) and SCNAs (median 4.25 × 10^−5^ fraction of genome altered), confirming high specificity of our algorithms (Supplementary Fig. 17). Our data suggest that WES of cfDNA provide similar SCNA results as ULP-WGS of cfDNA, exhibits very low false positive rates for SSNVs and SCNAs, and uncovers similar mutation rates compared to tumor biopsies.

**Fig. 2:**
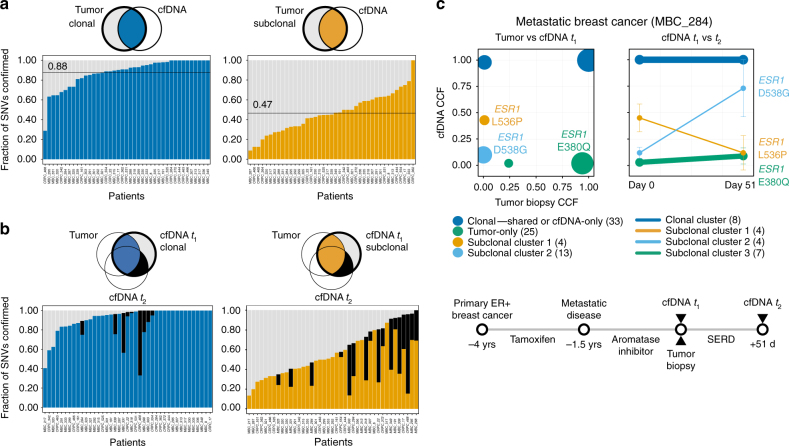
Comparison of whole-exome sequencing of cfDNA to whole-exome sequencing of matched tumor biopsies. **a** Fraction of clonal (≥0.9 cancer cell fraction, CCF) and subclonal (<0.9 CCF) SSNVs detected by MuTect in WES of tumor biopsies and confirmed (i.e., supported by ≥3 variant reads) in WES of cfDNA. Sites with <3 reads that had power <0.9 for mutation calling were not included when computing the fraction of SNVs confirmed (“Methods”). **b** Fraction of clonal and subclonal SSNVs detected in WES of cfDNA and confirmed in WES of tumor biopsies. For 18 patients with WES of cfDNA at a second time point *t*_2_, SSNVs not detected in the matched tumor biopsy but confirmed at *t*_2_ are indicated with black. **c** Analysis of clonal dynamics in an ER+ breast cancer patient diagnosed with metastatic disease 1.5 years (yrs) prior to biopsy and cfDNA collection (*t*_1_, Day 0). Clustering analysis of CCF for SSNVs between matched tumor biopsy and cfDNA (*t*_1_) is shown in the left panel. The right panel shows the CCF of four mutation clusters, one containing *ESR1* L536P (Subclonal Cluster 1, orange) and the other containing *ESR1* D538G (Subclonal Cluster 2, light blue), at *t*_1_ and *t*_2_ (51 days apart) from a patient with ER+ metastatic breast cancer being treated with a SERD. The lymph node biopsy was taken at the same time as cfDNA *t*_1_. Mutations were clustered by the CCFs for each pair of samples using Phylogic^39^ (“Methods”). Error bars represent the 95% credible interval of the joint posterior density of the clusters. Mutations, excluding indels, having ≥90% estimated power based on coverage in both samples are shown; clusters with fewer than three mutations are excluded. The number of mutations in each cluster is indicated in the legend in parentheses

We then examined the overlap of SSNVs and SCNAs between WES of cfDNA and matched tumor biopsies. We distinguished clonal and subclonal events by estimating the proportion of an observed somatic event out of the total tumor-derived DNA (cancer cell fraction, hereafter CCF) using ABSOLUTE^16^. We found, on average, 88% of the clonal (CCF ≥ 0.9; range 29–100%) and 47% of the subclonal (CCF < 0.9; range 9–100%) SSNVs that were detected in the tumor were confirmed to be present in cfDNA (i.e., supported by ≥3 variant reads, “Methods”) (Fig. 2a). Similarly, for SSNVs detected in the cfDNA, we found, on average, 88% of the clonal (range 33–100%) and 45% of the subclonal (range 14–88%) SSNVs were confirmed in the tumor (Fig. 2b). For 18 patients, we collected blood at a second time point (*t*_2_, 2–6 weeks later, Supplementary Data 7) and performed WES of cfDNA. We confirmed, on average, 56% of the subclonal SSNVs that were also detected in the earlier cfDNA sample (*t*_1_) but were not confirmed in the tumor biopsy (Fig. 2b). The confirmation of these cfDNA-exclusive events supports the possibility that these alterations may be derived from unprofiled tumor clones that were not captured by the core biopsy of a single lesion. We observed similar results for SCNA events of various sizes detected in the tumor (average 80% clonal, 77% subclonal confirmed in cfDNA) and detected in cfDNA (average 76% clonal, 70% subclonal confirmed in tumor) (Supplementary Fig. 18, Supplementary Data 4). Our findings suggest that cfDNA offers a suitable proxy for comprehensive genomic characterization of a tumor biopsy and may not derive solely from the single biopsied lesion.

Next, between cfDNA and the metastatic lesions (Supplementary Data 7), we observed a median of 46% (range 12–100%) of SSNVs (Supplementary Fig. 19, Supplementary Data 5) and 78% (range 25–95%) of genes altered by SCNAs (Supplementary Fig. 20, Supplementary Data 4) to be clonal (CCF ≥ 0.9) in both samples. For 17 of the patients with a second cfDNA sample, we observed clonal stability, with the majority (>50%) of SSNVs having similar clonality (±0.1 CCF) between time points (Supplementary Fig. 21, Supplementary Data 5). We also observed distinct subclonal patterns of SSNVs, including evolving clonal dynamics. For instance, in a metastatic breast cancer (MBC) patient (MBC_284) previously treated with an aromatase inhibitor, we detected multiple mutations in *ESR1* (D538G and L536P) in cfDNA at *t*_1_ (0.12 and 0.45 CCF) (Fig. 2c). Interestingly, the clonal fractions of these mutations were inverted at *t*_2_ (0.73 and 0.12, respectively) after 51 days of treatment with a selective estrogen receptor degrader (SERD), suggesting that these *ESR1* mutations may have different sensitivities to SERDs. We also detected an *ESR1* mutation (E380Q) in the tumor biopsy that was confirmed at low clonal fractions in cfDNA. These clonal shifts in resistance-associated mutations suggest that longitudinal analysis of WES of cfDNA may nominate potential mechanisms of resistance to therapy.

**Fig. 3:**
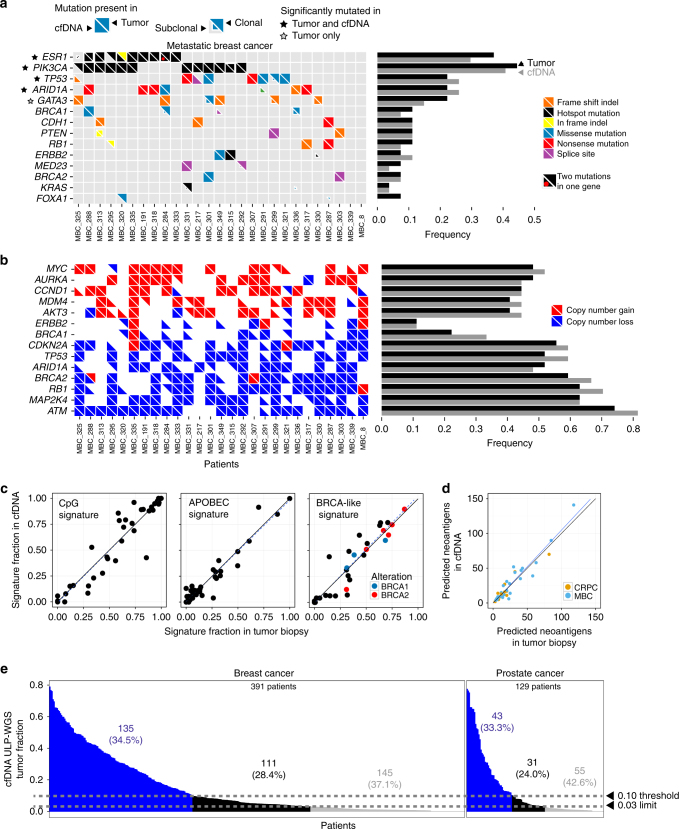
Genomic alterations of known significance and applicability to large cohorts. **a**, **b** The alteration status of significantly mutated genes predicted by MutSig2CV^23, 24^ (**a**), focal SCNAs (**b**), and known cancer-associated genes^22^ are shown for cfDNA and tumor biopsies from 27 metastatic breast cancer (MBC) patients. Mutated genes with MutSig2CV *q*-value < 0.1 are statistically significant. Mutations that were exclusively detected in one sample may be present at low CCF in the other matched sample but were excluded from the frequency calculation. SCNA frequencies were computed for oncogenes (*MYC* to *ERBB2*) and tumor suppressors (*BRCA1* to *ATM*) using only amplification and deletion status, respectively. Mutations were predicted using MuTect and SCNAs were predicted using ReCapSeg and ABSOLUTE. Red dot indicates distinct mutations in tumor and cfDNA. **c** Mutational signatures in whole-exome sequencing of cfDNA and tumor biopsies were predicted using a Bayesian non-negative matrix factorization (NMF) approach^29^ (“Methods”). Samples with predicted biallelic inactivation of *BRCA1/2* are indicated in red and blue. Black line denotes *y* = *x*; blue line denotes model fit using linear least squares regression. **d** Neoantigen burden, defined as the number of predicted neoantigen SSNVs, was calculated using NetMHCpan^33^ (“Methods”). Black line denotes *y* = *x*; blue line denotes model fit using linear least squares regression. **e** Applicability to many patients with metastatic cancer. Tumor fractions estimated from ULP-WGS of cfDNA from 903 blood samples from 391 patients with metastatic breast cancer and 536 blood samples from 129 patients with metastatic prostate cancers. The earliest blood drawn for each patient is shown. Samples with coverage <0.05× were excluded

We then assessed whether WES of cfDNA can serve as a proxy for tumor biopsies in multiple applications of cancer exome analyses. First, we compared known cancer-associated somatic alterations^22^ between cfDNA and tumor biopsies for 27 metastatic breast and 14 metastatic prostate cancer patients (Supplementary Data 8). In breast cancer, we observed similar frequencies of altered genes (Pearson’s *r* = 0.97) in both cfDNA and tumor biopsies, including mutations in *TP53*, *ESR1*, and *PIK3CA*, amplifications of *MYC*, *CCND1*, *ERBB2*, *PIK3CA*, and losses of *ATM* and *RB1* (Fig. 3a). Similarly, in prostate cancer, we observed frequent amplifications of *AR* as well as mutations and LOH of *TP53* (Supplementary Fig. 22). Next, to discover statistically significant genes recurrently mutated above background rates, we applied MutSig2CV^23, 24^ independently to cfDNA and tumor biopsies and identified *ESR1, TP53, PIK3CA, ARID1A* (Fig. 3a, Supplementary Data 8). Among these mutated genes, we found a statistically significant enrichment of non-silent mutations in *ESR1* and *ARID1A* for both cfDNA and tumor biopsies in 20 ER+/HER2− metastatic cancer patients when compared to 279 primary ER+/HER2− breast carcinomas published previously by The Cancer Genome Atlas (TCGA)^25^ (Bonferroni-corrected Fisher’s exact test, *p* = 1.46 × 10^−8^ and 2.58 × 10^−2^ respectively). The metastatic breast cancer biopsies in this study are derived from a larger cohort in which *ARID1A* was found to be significantly mutated and enriched with respect to TCGA^26^. The mutational enrichment was significant in both metastatic biopsies and cfDNA, suggesting that cfDNA exome sequencing can lead to similar biological insights as tumor biopsies and may enable genomic discovery from larger cohorts.

### Mutation signatures and neoantigens can be detected in cfDNA

As mutational processes operating in tumors have been associated with potential sensitivity to specific therapies^27^ and their detection in cfDNA could be clinically significant, we analyzed the mutational signatures^28, 29^ present in cfDNA and tumor biopsy. We identified three previously^30^ described mutational signatures associated with aging (C>T mutations at CpG dinucleotides), APOBEC activity (C>T or C>G at a TC[A/T] context), and DNA homologous recombination deficiency (*BRCA*-like^27^) (Fig. 3c, Supplementary Fig. 23, “Methods”). We found that the predicted fraction of mutations belonging to each signature was highly concordant between cfDNA and tumor biopsies (adjusted *R* ^2^ = 0.92, *p* < 1 × 10^−16^, Fig. 3c). We also observed that patients with predicted biallelic inactivation of *BRCA1* or *BRCA2* had higher *BRCA*-like signature activity in both cfDNA and tumor biopsies (Wilcoxon rank-sum test, one-tailed, *p* < 0.01). These results suggest that analysis of cfDNA may be a complementary approach to predict homologous recombination deficiency and could provide information regarding potential sensitivity to drugs, such as PARP inhibitors^31^ that target this pathway.

Furthermore, as cancer immunotherapies have been effective in clinical trials and analysis of neoantigens may influence treatment strategies^32^, we compared the number of somatic mutations that were predicted to be neoantigens in cfDNA and matched tumor biopsies. We predicted the binding affinity of missense SNVs to patient-specific MHC Class I alleles inferred from germline WES data^33, 34, 35^, and considered any mutation with an IC50 < 500 nM to be a predicted neoantigen (“Methods”). We found that the number of predicted neoantigens was strongly correlated between cfDNA and tumor biopsies (adjusted *R* ^2^ = 0.90, *p* < 1 × 10^−16^), suggesting that WES of cfDNA could lead to similar prediction of potential tumor immunogenicity as would sequencing of tumor biopsies (Fig. 3d).

### cfDNA exome sequencing is feasible in advanced cancer patients

Finally, our results indicate that many patients with metastatic cancer will have sufficient tumor-derived cfDNA for WES. We analyzed ULP-WGS of cfDNA from 903 blood samples from 391 patients with metastatic breast cancer and 536 blood samples from 129 patients with metastatic prostate cancer (Fig. 3e, Supplementary Fig. 24, Supplementary Data 1). Overall, we found 73% of patients with metastatic breast and prostate cancer, had detectable (≥0.03) tumor-derived cfDNA. Considering only the earliest blood draw from each patient, 34.5% and 33.3% of breast and prostate cancer patients, respectively, had sufficient tumor fraction (≥0.1)^11, 16^ for standard WES (Fig. 3e). Additionally, when considering all blood samples, 43 and 49% of breast and prostate cancer patients had at least one sample with ≥0.1 tumor fraction (Supplementary Fig. 24). Subsequent analysis of SCNAs detected from ULP-WGS of these samples revealed SCNA landscapes that closely reflected those reported^36^, including biopsies of metastatic tumors from 150 patients with castration-resistant prostate cancer (CRPC)^20^ (Supplementary Fig. 24, Supplementary Data 4). We also identified frequent alterations of known tumor suppressor genes (e.g., *ATM, RB1, TP53, CDKN2A/B, PTEN*, and *PPP2R2A*) and oncogenes (e.g., *CCND1, AKT1, GATA3, ERBB2, PIK3CA*, and *AR*)^20, 36, 37^. Our results demonstrate that WES is possible in a substantial fraction of patients with metastatic breast and prostate cancers. Furthermore, using the estimated tumor fraction can help to calibrate the required sequencing depths for lower tumor content samples (Supplementary Fig. 25).

## Discussion

Our study has overcome three major hurdles for making WES of cfDNA a routine possibility for patients with metastatic cancer: (1) efficient screening for tumor content prior to WES; (2) comprehensive benchmarking of cfDNA and conventional biopsies; (3) applicability to many patients with metastatic cancer. While many studies have emphasized targeted sequencing of cfDNA, we have established feasibility for reproducible and scalable profiling of whole cancer exomes from cfDNA. Our characterization of 41 pairs of cfDNA and tumor whole-exomes constitutes by far the most comprehensive comparisons reported to date; the first to examine clonal relationships in SSNVs and SCNAs between cfDNA and tumor biopsies using WES; and the first to analyze significantly mutated genes, mutational signatures, and neoantigens among cohorts of patients using WES of cfDNA.

The differences in SSNVs and SCNAs between cfDNA and tumor biopsies may be attributable to location and timing of biopsy, differential release of cfDNA among lesions, and extent of tumor heterogeneity^38, 39^ within a patient. It is also possible that nucleosome positioning^18^ or epigenetic modifications^40^ may affect the ability to detect certain somatic alterations in cfDNA. Further, the assessment of clonality in cfDNA may be confounded by the contribution of cfDNA derived from multiple metastases. Nonetheless, WES enables comprehensive clonal analysis of cfDNA to track tumor evolution and identify mechanisms of resistance to targeted therapy. While further investigation is required to determine the feasibility of profiling cfDNA from patients with earlier stages of disease, the ability to detect SCNAs and estimate tumor content from ULP-WGS alone may have roles in broader efforts to study aneuploidy and routine clinical monitoring of metastatic disease.

We had previously demonstrated feasibility for whole-exome sequencing of circulating tumor cells^41^. Here we established an analogous approach for whole-exome sequencing of cfDNA. Together, these two approaches may unlock routine and comprehensive genomic characterization of types and stages of cancer that are infrequently biopsied in clinical practice.

## Methods

### Human subjects

Patients with metastatic breast cancer (MBC) were prospectively identified for enrollment into tissue analysis and banking cohorts (Dana-Farber Cancer Institute IRB protocol identifiers 05-246, 09-204, 12-431 [NCT01738438; Closure effective date 6/30/2014]). Eligible patients included those with known metastatic breast cancer as well as those with newly diagnosed breast cancer (de novo metastatic disease). After obtaining informed consent for genomic analysis of their blood and/or tumor tissue, an initial blood draw was collected. Accessible metastatic, non-bone sites (e.g., breast, skin, and lymph node) were preferentially identified for biopsy. When feasible, a corresponding blood draw for plasma was performed within 7 days of a metastatic tumor biopsy. A subset of patients underwent subsequent blood draws at the time of treatment switch, 4–6 weeks after treatment switch, and every 3 months if on stable treatment.

Eligible metastatic CRPC patients were identified through the Prostate Clinical Research Information System (CRIS) database at Dana-Farber Cancer Institute^42^. The CRIS system comprises data-entry software, a central data repository, collection of patient data including comprehensive follow-up of all patients, and tightly integrated security measures, as previously described^42^. All patients provided written informed consent to allow the collection of tissue and blood and analysis of clinical and genetic data for research purposes (DFCI Protocol # 01-045, IRB expiration date 01/13/2017). The cohorts accrued to this study were patients who either (1) were identified based on prospective chart review to have PSA >20 ng/mL, progressive disease based on rising PSA, and scan progression; (2) were participants in a Phase I study of crizotinib in combination with enzalutamide (DFCI Protocol # 14-230, IRB expiration date 05/05/2017, NCT02207504) or a Phase Ib study of abiraterone in combination with ARN-509 (DFCI Protocol # 12-338, IRB expiration date 08/20/2016, NCT01792687); or (3) were eligible for metastasis biopsy after progression on enzalutamide or abiraterone through the Stand Up 2 Cancer/PCF Dream Team Effort based on participation in one of the following protocols: a Phase II study of abiraterone in combination with dutasteride (DFCI Protocol # 10-448, IRB expiration date 12/30/2016, NCT01393730), a Phase II trial of enzalutamide with correlative assessment of AR signaling (DFCI Protocol # 13-301, IRB expiration date 07/30/2016, NCT01942837), a Phase II trial of abiraterone without exogenous glucocorticoids (DFCI Protocol # 13-449, IRB expiration date 10/01/2016, NCT02025010), and a tumor biopsy protocol to assess tissue correlates of therapeutic response (DFCI Protocol # 09-171, IRB expiration date 09/14/2016). Blood specimens were prospectively collected from eligible patients.

Fresh whole blood (10–20 cc) from appropriately consented healthy donors was obtained through Research Blood Components (http://researchbloodcomponents.com/services.html). The donor cohort was comprised of healthy males and females between 18 and 65 years of age. Additional plasma samples were obtained from appropriately consented healthy individuals under DFCI Protocol # 03-022, IRB expiration date 12/28/2017).

### Clinical specimens

Venous blood samples (10 cc) were collected in EDTA (BD) or CellSave Preservative (Cell Search) tubes. Tubes were processed within 4 h of collection by freezing of a small aliquot (610 µL) of whole blood at −80 °C and centrifuging the remaining whole blood at 1000–1900 × *g* for 10 min at room temperature. After discarding the red blood cells and buffy coat, plasma was centrifuged a second time at 15,000 × *g* for 10 min at room temperature in low-bind tubes to remove residual cells from plasma. Supernatants were then frozen at −80 °C until ready for further processing.

Matched tumor biopsies were processed and sequenced through the Broad Institute Genomics Platform’s Research Whole Exome Sequencing deep coverage pipeline (http://genomics.broadinstitute.org/data-sheets/DTS_WES_1Page_5-2016_0.pdf). For 1× whole-genome sequencing of 22 patient tumor biopsies, 25 ng genomic DNA was subjected to the library construction steps of the Nextera Rapid Capture Exome Kit (Illumina) at half volume but not to hybrid selection. Sequencing to generate 100 bp paired-end reads was performed on the Illumina HiSeq2500 in rapid-run mode.

### Extraction and quantification of cfDNA

Frozen aliquots of plasma were thawed at room temperature. cfDNA was extracted from 1 to 7 mL of plasma and eluted into 40–80 µL of re-suspension buffer using the Qiagen Circulating DNA kit on the QIAsymphony liquid handling system. Extracted cfDNA was frozen at −20 °C until ready for further processing. Quantification of extracted cfDNA was performed using the PicoGreen (Life Technologies) assay on a Hamilton STAR-line liquid handling system.

### Extraction and quantification of germline DNA

Whole blood was thawed at room temperature. Germline DNA was extracted from 400 µL of blood and eluted into 200 µL of re-suspension buffer using the Qiasymphony DSP DNA midi kit on the QIAsymphony liquid handling system. Samples were then frozen at −20 °C until ready for further processing. Extracted gDNA was quantified using the PicoGreen (Life Technologies) assay on a Hamilton STAR-line liquid handling system.

### Library construction and sequencing of cfDNA

Library construction of cfDNA was performed using the Kapa Hyper Prep kit with custom adapters (IDT and Broad Institute). A total of 5–20 ng of cfDNA input was used for ULP-WGS. A Hamilton STAR-line liquid handling system was used to automate and perform this method. Constructed sequencing libraries were pooled (2 µL of each × 96 per pool) and sequenced using 100 bp paired-end runs over 1× lane on a HiSeq2500 (Illumina) for ULP-WGS.

When possible, 20 ng of cfDNA input was used to construct another cfDNA library for WES, which afforded greater library complexity and reduced the depth of sequencing required to achieve the desired mean target coverage. Library construction was performed using the Kapa Hyper Prep kit with custom adapters (IDT and Broad Institute) on a Hamilton STAR-line liquid handling system. Libraries were then quantified using the PicoGreen (Life Technologies) assay on a Hamilton STAR-line liquid handling system and pooled up to 12-plex. Hybrid selection of cfDNA libraries was performed using the Nextera Rapid Capture Exome kit (Illumina) with custom blocking oligos (IDT and Broad Institute). Sequencing to generate 100/101 bp paired-end reads was performed on the Illumina HiSeq2500/HiSeq4000 in high-output mode with two to four libraries per lane. Out of the 1684 cancer patient cfDNA samples collected, we successful constructed libraries and sequenced 1596 samples.

For deeper whole-genome sequencing, we used the best possible libraries constructed with 5–20 ng of cfDNA input. Re-sequencing to greater depths (10×–48×) with 100/101 bp paired-end reads was performed on Illumina HiSeq2500/HiSeq4000 in high-output mode.

### Extraction and sequencing of genomic DNA

For whole-exome sequencing, DNA Library construction and hybrid selection of gDNA was performed using the Nextera Rapid Capture Exome kit (Illumina) at half volume with 25 ng of DNA input. Sequencing was performed on the Illumina HiSeq2500 in high-output mode with 100 bp paired-end reads. Four to six libraries were pooled per lane.

### Analysis of ULP-WGS of cfDNA using ichorCNA

In order to assess the presence of detectable tumor DNA, we performed ULP-WGS of cfDNA to an average genome-wide fold coverage of ∼0.1×. We analyzed the depth of coverage in a ULP sample to evaluate large-scale copy number alterations (CNAs) and aneuploidies. We developed a probabilistic model and implemented a software package called ichorCNA, which uses concepts from existing algorithms^17, 43^ designed for deep coverage WGS/WES data to simultaneously predict regions of CNAs and estimate the fraction of tumor in ULP-WGS. We applied ichorCNA to analyze 1596 metastatic breast (974) and prostate (622) cancer cfDNA samples, and 27 healthy donor cfDNA samples. Samples with genome-wide coverage <0.05 × were excluded (71 MBC, 86 CRPC, 0 HD). The workflow consists of three steps: (1) Computing read coverage, (2) data normalization, and (3) CNA prediction and estimation of tumor fraction. Below, we describe the challenges, model assumptions, analysis workflow, and the probabilistic model. The ichorCNA software can be obtained at https://github.com/broadinstitute/ichorCNA.

### Challenges of ULP-WGS of cfDNA and ichorCNA assumptions

ULP-WGS of cfDNA presents several analytical challenges including (a) very low coverage of sequencing; (b) absence of matched normal germline DNA; and (c) low tumor content of many cfDNA samples. Therefore, we implemented a solution that accounts for these challenges using several assumptions that will help with the analysis and interpretation. (1) Large-scale CNA can be detected by evaluating read coverage in large, equal-sized genomic windows (or bins). (2) Homozygous deletions are typically at smaller scales than the large bin sizes used here and are not considered. (3) Clonal copy number states should be discrete integers. (4) This bin size is large enough to overcome any biases related to nucleosome positioning which is at the scales of 166 bp and 332 bp. (5) Due to the low coverage and absence of allelic information, only one subclone is assumed to be detectable. The last assumption is a consequence of a limitation of the algorithm to reliably and explicitly distinguish large numbers of subclones, given the low coverage and low tumor content.

### ichorCNA: analysis workflow

The genome is divided into *T* non-overlapping windows, or bins, of 1 Mb. Aligned reads are counted based on overlap within each bin. This was done using the tools in HMMcopy Suite^43^ (http://compbio.bccrc.ca/software/hmmcopy/). Centromeres are filtered based on chromosome gap coordinates obtained from UCSC for hg19, including one 1 Mb bin up- and downstream of the gap.

The short fragment sizes of cfDNA (e.g., 166 bp) often contain overlapping paired reads for 100 bp read lengths and can lead to two overlapping reads representing a single fragment. Abundance of cfDNA fragments has been shown to exhibit tissue-specific differences along local ~200 bp scale regions of the genome^18^. For this analysis, because read counts are computed for large bins, the double-counting at ~200 bp scale is not likely to have a major effect.

The read counts are then normalized to correct for GC-content and mappability biases using HMMcopy R package^43^. Briefly, two LOESS regression curve-fitting are performed to the bin-wise (1) GC-fraction and read counts, followed by (2) mappability uniqueness score and read counts. The curve-fitting was only applied to autosomes. This generates corrected read counts *r*_*t*_ for each bin $t \in \left\{ {1, \ldots ,T} \right\}$.

Next, the gender of the patient is determined by inspecting the corrected read counts in chromosome X and Y. We used two criteria to determine if the sample is a male (otherwise the sample is a female): (1) the proportion of uncorrected chrY read counts out of the total number of reads is >0.001 and (2) the median corrected log ratio of chrX is <−0.5. If the sample is a male, then the bins in chrX are re-scaled, ${r_{t \in {\rm{chrX}}}}/{\rm{median}}\left( {{r_{t \in {\rm{chrX}}}}} \right)$.

We also performed ULP-WGS on cfDNA from 27 healthy donors using the same protocol in order to create a reference data set. These data help to further normalize the cancer patient cfDNA to correct for systematic biases arising from library construction, sequencing platform, and cfDNA-specific artifacts. We computed the median at each bin across the 27 samples to generate a reference data set, ${h_{1:T}}$. For a given cancer patient cfDNA sample and each bin $t$, the ${\rm{lo}}{{\rm{g}}_2}$ copy ratios are computed as ${l_t} = {\rm{lo}}{{\rm{g}}_2}\left( {\frac{{{r_t}}}{{{{\bar h}_t}}}} \right)$.

### ichorCNA: copy number prediction and tumor fraction estimation

The cancer patient cfDNA CNA signal can be represented as an admixture between DNA fragments derived from tumor and non-tumor cells. We use a two-component mixture to model this explicitly^16, 17, 43–45^

1 $${\rm{observed}}\,{\rm{CNA}} \propto 2n + \left( {1 - n} \right)c$$

where *n* is the non-tumor proportion, (1−*n*) is the tumor proportion, and *c* is the copy number for a specific alteration (e.g., one for deletion, three for gain, etc.). For subclonal events, a third component is used to represent DNA fragments derived from tumor cells not harboring the CNA event^16, 17, 45^.

2 $${\rm{observed}}\,{\rm{subclonal}}\,{\rm{CNA}} \propto 2n + 2s\left( {1 - n} \right) + \left( {1 - s} \right)\left( {1 - n} \right)c$$

where *s* is the proportion of tumor not containing the event with *c* copy number. Thus, (1−*s*) is similar to the definitions of tumor-cellular-prevalence^17^ or cancer-cell-fraction^16^ for tissue tumors.

*State model*. The copy number states are mapped to hemizygous deletions (HETD, 1), copy neutral (NEUT, 2), copy gain (GAIN, 3), amplification (AMP, 4), and high-level amplification (HLAMP, 5–7 copies). The homozygous deletions state (HOMD, 0 copies) is excluded because the analysis is focused on large-scale multiple mega-bases per event. For the analysis performed in this study, we fixed the copy number to be ${K_{{\rm{clonal}}}} = \left\{ {1,2,3,4,5} \right\}$ For subclonal events, two additional states are included: subclonal hemizygous deletion (HETD_sc_) and subclonal copy gain (GAIN_sc_),

$$K = \left\{ {{K_{{\rm{clonal}}}},{{\left\{ {1,3} \right\}}_{{\rm{subclonal}}}}} \right\}.$$

A copy number state is assigned to *G*_*t*_ for each bin *t* and the initial distribution of these copy number states is given by ${G_0} \sim {\rm{Mult}}\left( \pi \right)$.

ichorCNA uses a hidden Markov model (HMM) to predict segments of CNAs and to estimate the tumor fraction from ULP-WGS of cfDNA. Details of the Bayesian statistical framework of the hidden Markov model and its inference using the expectation–maximization (EM) algorithm are described next.

*Emission model*. The input log copy ratios *l*_1:*T*_ is modeled using a Student’s *t*-distribution with *μ*_*g*_, *λ*_*g*_, and *v*_*g*_ as the mean, precision, and degrees of freedom, conditional on copy number state $g \in K$ at bin *t*,

$$p\left( {\left. {{l_t}} \right|{G_t} = g} \right) = {\rm{St}}\left( {\left. {{l_t}} \right|{\mu _g},{\lambda _g},{\nu _g}} \right)$$

Mean *μ*_*g*_ is defined by the three-component mixture (Eq. (2))^17, 45^ for copy number state *g* with unknown global parameters *n* and average tumor ploidy *ϕ*,

$${\mu _g} = {\rm{log}}\left( {\frac{{2n + 2s\left( {1 - n} \right) + \left( {1 - s} \right)\left( {1 - n} \right){c_g}}}{{2n + \left( {1 - n} \right)\phi }}} \right)$$

For clonal copy number states, since *s* = 0 then *μ*_*g*_ is defined by the two-component mixture (Eq. (1)),

$${\mu _g} = {\rm{log}}\left( {\frac{{2n + \left( {1 - n} \right){c_g}}}{{2n + \left( {1 - n} \right)\phi }}} \right)$$

Precision *λ*_*g*_ for each $g \in K$ are also model parameters. The degrees of freedom *v*_*g*_ is a constant (2.1) and is not estimated.

*Transition model*. A stationary (homogeneous) transition model is used in the HMM. Because all bins have equal-sized intervals, with the exception of centromere regions, a non-stationary transition model to account for varying genomic distances^17^ between data points was not used. The transition matrix containing the transition probabilities is given by

$$\begin{array}{ccccc}\\ & p\left( {{G_t} = j \, \left| {{G_{t - 1}}} \right. = i} \right) = {A_{ij}}\\ \\ & {A_{ij}} = \left\{ {\begin{array}{*{20}{l}} e \hfill & {i = j} \hfill \\ {\frac{{1 - e}}{{\left| {K }\right|- 1 }}} \hfill & {{\rm{otherwise}}} \hfill \\ \end{array}} \right.\\ \end{array}$$

where *e* is set to 0.99999.

*Prior model*. The HMM is implemented as a Bayesian framework with priors for each model parameter: Student’s *t* parameters *μ*_*g*_, *λ*_*g*_ for each $g \in K$, transition probabilities *A*, initial state distribution *π*, and global parameters *n*, *s*, and *ϕ*,

$$\begin{array}{*{20}{l}}\\ n \sim {Beta \, \left( {{\alpha _n},{\beta _n}} \right)} \hfill \\ s \sim{Beta \, \left( {{\alpha _s},{\beta _s}} \right)} \hfill \\ \phi \sim {Gamma \, \left( {{\alpha _\phi },{\beta _\phi }} \right)} \hfill \\ {{\lambda _g}} \sim {Gamma \, \left( {{\alpha _g},{\beta _g}} \right)} \hfill \\ A \sim {{\rm{Dir}} \, \left( {{\delta _A}} \right)} \hfill \\ \pi \sim {{\rm{Dir}} \, \left( {{\delta _{pi}}} \right)} \hfill \\ \end{array}$$

where $\psi = \left\{ {{\delta _A},{\delta _\pi },{\alpha _g},{\beta _g},{\alpha _n},{\beta _n},{\alpha _s},{\beta _s},{\alpha _\phi },{\beta _\phi }} \right\}$ for all $g \in K$ are the hyper-parameters.

Hyperparameter values are set to represent uniform priors. However, for the prior of the Student’s *t* precision, *α*_*g*_ = 3 and ${\beta _g} = {\left( {\frac{{{\rm{sd}}\left( {{l_{1:T}}} \right)}}{{\sqrt {\left| K \right|} }}} \right)^{ - 1}}$ where ${\rm{sd}}\left( {{l_{1:T}}} \right)$ is the standard deviation of log ratio data to reflect the variance in the specific sample.

*Learning and inference*. The model parameters $\theta = \left\{ {{\mu _{1:\left| K \right|}},{\lambda _{1:\left| K \right|}},A,\pi ,n,\phi } \right\}$ are estimated using the EM algorithm given the data ${\cal D} = \left\{ {{l_{1:T}}} \right\}$. In the E-step, we applied the forwards–backwards algorithm to compute the posterior probabilities, $p\left( {{G_t} = g\left| {D,\theta } \right.} \right)$. In the M-step, the parameters *θ* ^(*n*)^ at EM iteration *n* are estimated using the maximum a posteriori (MAP) estimate,

$${\theta ^{\left( n \right)}} = {\rm{argma}}{{\rm{x}}_\theta }\left\{ {p\left( {\left. G \right|D,{\theta ^{\left( {n - 1} \right)}}} \right)p\left( {D,\left. G \right|{\theta ^{\left( n \right)}}} \right)} \right\}$$

The converged parameters $\hat \theta$ are determined by the EM convergence criteria such that the complete-data log-likelihood (including priors)

$${F^{\left( n \right)}} = {\rm{log}}\,p\left( {D,Z\left| {{\theta ^{\left( {n - 1} \right)}}} \right.} \right) + {\rm{log}}\,p\left( {{\theta ^{(n)}}\left| \psi \right.} \right)$$

changes <0.1% $\left( {{F^{\left( n \right)}} - {F^{\left( {n - 1} \right)}} < 0.001} \right)$. The complete-data log-likelihood at convergence is denoted $\hat F$.

We then apply the Viterbi algorithm to find the optimal copy number state path for all bins,

$${\hat G_{1:T}} = {\rm{argma}}{{\rm{x}}_G}\left\{ {p\left( {{G_{1:T}}\left| {D,\hat \theta } \right.} \right)} \right\}$$

Chromosome 19 shows systematic decrease in log_2_ copy ratio values across majority of samples for bins within chr19 after GC-content correction, including cancer patient and healthy donor cfDNA. Because the healthy donor samples were used for genome-wide normalization, the chromosome 19 bias, along with other systematic large-scale biases are accounted for. Regardless, we still excluded chromosome 19 during parameter estimation (i.e., EM) since it is negligible number of data points in the learning. However, chromosome 19 is still included in the Viterbi algorithm as part of generating a genome-wide solution.

In order to avoid the local optimal limitation of EM, we perform multiple restarts by performing EM over a range of initializations for normal fraction (${n^{\left( 0 \right)}} \in \left\{ {0.35,0.45,0.50,0.65,0.75,0.85,0.95} \right\}$) and tumor ploidy (${\phi ^{\left( 0 \right)}} \in \left\{ {2,3,4} \right\}$) parameters. The solution with the maximum complete-data log-likelihood, $\hat F$, over each initialization pair, $\left( {{n^{\left( 0 \right)}},{\phi ^{\left( 0 \right)}}} \right)$ is chosen.

Due to the problem of identifiability between clonal and subclonal events, which is especially challenging in ULP sequencing and the absence of allelic information, solutions with >50% of the genome harboring subclonal CNA or >70% of CNA calls being subclonal are not selected. Further, solutions with a total alteration fraction (based on bins) <0.05 and having the largest CNA event be <50 bins are assigned a tumor fraction of zero.

### ichorCNA: code availability and run-time

ichorCNA is implemented as an R package and can be obtained at https://github.com/broadinstitute/ichorCNA.

The ichorCNA HMM component has complexity ${\cal O}\left( {{\rm{KT}}} \right)$ in memory and ${\cal O}\left( {{{\rm{K}}^2}{\rm{T}}} \right)$ in time. The run-time of the algorithm for 0.1× coverage is on the order of 1 min for read coverage computation and 1 min for analysis using the HMM.

### ichorCNA benchmarking and performance evaluation

In order to determine the performance of the algorithm for predicting CNA and estimating tumor fractions for ULP-WGS, we generated four benchmarking data sets from real cancer patient cfDNA sequencing to evaluate ichorCNA in a controlled manner.

1. “Serial” mixtures at varying tumor fraction and coverage (0.01×–1×) using the whole genome of a breast cancer patient cfDNA sample (MBC_288).
2. “Merged” mixtures at 0.1× coverage generated using 44 breast/prostate and 18 healthy donor cfDNA ULP-WGS samples with ≥0.05×.
3. “Exact tumor fraction” mixtures at 0.1× coverage ranging from 0.1 to 1.0 (0.1 increments) tumor fraction using 50 breast/prostate cfDNA ULP-WGS samples.
4. “Spike-in” analysis to determine the minimum number of CNAs, size, and magnitude to detect tumor-derived DNA using a subset of 10 cancer patient cfDNA samples.

For copy number prediction evaluation, binary classification metrics of precision (positive predictive value; $\frac{{{\rm{TP}}}}{{{\rm{TP}} + {\rm{FP}}}}$), recall (sensitivity; $\frac{{{\rm{TP}}}}{{{\rm{TP}} + {\rm{FN}}}}$), and *F*-measure (F1-score; $\frac{{2 \times {\rm{precision}} \times {\rm{recall}}}}{{{\rm{precision}} + {\rm{recall}}}}$), were computed separately for copy number gains and deletions. True positives (TP) are defined as the number of bins in which the copy number is < 2 or >2 for deletion and amplifications, respectively, observed in both the admixture sample and the ground truth.

*Serial mixtures at varying coverage (0.01×–1×)*. First, we sequenced the whole genome of a breast cancer patient cfDNA sample (MBC_288) to ~10× coverage. We analyzed this sample using the same algorithm for ULP-WGS data and determined the CNA events and tumor fraction to be 0.78 (Supplementary Fig. 2). Both the CNA events and tumor fraction estimate were further confirmed in analysis of the WES of the same sample using TITAN^17^ and ABSOLUTE ^16^ (Supplementary Fig. 15, Supplementary Data 4). Based on these results, we used the 10× WGS data for down-sampling and admixing experiments, and using the 10× ULP-WGS results as ground truth. We also performed WGS on cfDNA of a healthy donor (HD_2) to 10× coverage to use as the normal sample to admix in the experiment.

We generated tumor-normal admixtures ranging from 0.01 to 0.21 (0.02 increments) and 0.25 to 0.45 (0.05 increments) of sample MBC_288 and 0.99 to 0.79 (0.02 increments) and 0.75 to 0.55 (0.05 increments) of sample HD_2 such that the two proportions summed to one. For each admixture, we also down-sampled to coverages of 0.01×, 0.05×, 0.10×, 0.20×, 0.30×, 0.40×, 0.50×, 0.60×, 0.70×, 0.80×, 0.9×, 1.0×. The down-sampling and mixing was done using Picard *DownsampleSam* and using the *PROBABILITY* argument. In the end, a total of 192 down sampled admixture samples were generated.

We applied the ULP-WGS analysis to call copy number and estimate tumor fraction on each sample independently. We used the same procedure as described above, including using 1 Mb bins, with the exception that we did not use the *ϕ* = {3,4} for initializations during EM restarts.

*Merged* ×*0.1 mixtures*. For 44 breast/prostate cfDNA (CT) ULP-WGS samples with ≥0.05× coverage (1.5 million reads) and matching WES samples, we downsampled to ~0.05× coverage. We also down-sampled 18 healthy donor ULP-WGS samples to ~0.05× coverage (Supplementary Fig. 12). Next, we merged each of the 44 downsampled cfDNA CT samples with each of the 18 healthy donor samples to generate 792 mixtures at ~0.1× coverage with an expected tumor fraction, ${\rm{expTF}} = \frac{{\# \,{\rm{reads}}\,{\rm{CT}}}}{{\# \,{\rm{reads}}\,{\rm{CT}} + \# \,{\rm{reads}}\,{\rm{HD}}}} \times {\rm{WES}}\,{\rm{purity}}$, where ${\rm{WES}}\,{\rm{purity}}$ is the estimated tumor purity of the CT sample from WES using ABSOLUTE/TITAN. The rounded ploidy estimate from ABSOLUTE/TITAN was used for the ploidy initialization in the analysis on the mixtures. Duplicates have been removed prior to down-sampling as to not skew the expected tumor fraction. Only autosomes 1–22 were considered.

*Exact tumor fraction (0.01 to 0.1) mixtures at 0.1× coverage*. For 50 breast/prostate cfDNA (CT) ULP-WGS samples with a matching WES sample, we down-sampled to the number of reads required to reach exactly 0.01, 0.02, …, 0.09, 0.10 tumor fraction at 0.1× coverage (Supplementary Fig. 13). We used a deeper sequenced healthy donor (HD_2) to dilute these mixtures. The number of reads required for a CT sample *S* at a specific tumor fraction ${\rm{TF}} \in \left\{ {0.01,0.02,...,0.09,0.10} \right\}$ was computed as ${\rm{CT}}\_{\rm{reads}}\left( {S,{\rm{TF}}} \right) = \frac{{1.5 \times {{10}^6}\,{\rm{reads}}\,*\,{\rm{TF}}}}{{{\rm{WES}}\,{\rm{purity}}}}$. The number of reads required for HD_2 were computed as ${\rm{HD}}\_{\rm{reads}}\left( {S,{\rm{TF}}} \right) = 1.5 \times {10^6} - {\rm{CT}}\_{\rm{reads}}\left( {S,{\rm{TF}}} \right)$. ${\rm{WES}}\,{\rm{purity}}$ is the estimated tumor purity of the CT sample from WES using ABSOLUTE/TITAN. The rounded ploidy estimate from ABSOLUTE/TITAN was used for the ploidy initialization in the analysis on the mixtures. The total number of mixtures generated was 496 (instead of 500) because one CT sample did not have sufficient coverage for mixtures >0.6×. Duplicates have been removed prior to downsampling as to not skew the expected tumor fraction. Only autosomes 1–22 were considered.

*Spike-in analysis to determine the minimum CNA event number, size, and magnitude to detect tumor-derived DNA*. For 10 of the cancer patients from the “exact tumor fraction mixture” experiment, we took each of the ten mixtures (0.01–0.1 tumor fraction) for each of the patients and simulated a copy neutral genome with (a) a single loss, (b) a single gain, or (c) a loss and a gain of varying lengths. Using 10 different patient samples allows for analysis of the variance in the experiment. The simulation of copy neutral data was sampled from the data of true copy neutral of the patient’s original profile. Likewise, the spike-in of gains and/or losses was generated using data from the patient’s original profile so that the inherent sample-specific variance is maintained. An example of these spike-in data sets is shown in Supplementary Fig. 14.

### ichorCNA lower limit of tumor detection in cfDNA

The lower limit of detection of 0.03 was determined using several analyses. First, the maximum absolute error across all coverages ≥0.10× and all admixture proportions was ≤0.03. Next, using the “merged” 0.1× mixtures (792 samples; Supplementary Fig. 12) and “exact tumor fraction” 0.1× mixtures (496 samples; Supplementary Fig. 13), we assessed the sensitivity and specificity for correctly classifying the presence or absence of tumor DNA. At a threshold 0.03 tumor fraction, the sensitivity of correctly predicting the presence of tumor is 95% (out of 1288 positives) while the specificity for correctly predicting the absence of tumor is 91% (out of 22 healthy donors) (Supplementary Data 3). A similar analysis was performed to evaluate the decision threshold of 0.1 tumor fraction, achieving 91% sensitivity (presence of tumor) and 100% specificity (absence of tumor).

### Comparison of cfDNA ULP-WGS to other data types

*Comparison with deeper coverage WGS (>10× coverage)*. Having established the performance from controlled benchmarking comparisons, we performed deeper coverage (>10×, range 10–48×) WGS for seven of the cfDNA samples. We compared the log_2_ copy ratios and copy number between the datatypes, using CNA predicted from the deeper WGS using TITAN as ground truth. For the log_2_ copy ratio comparison, we computed the Spearman correlation at 1 Mb bins for both datatypes (Supplementary Fig. 3). For the CNA performance, we used TITAN CNA predictions, which were generated using 1 Mb genome-wide log_2_ copy ratios and exonic heterozygous SNPs determined from the WES matched normal sample (since WGS for matched normals were not available, Supplementary Fig. 5a).

*Comparison between cfDNA ULP-WGS to matched tumor biopsy (WES and 1× WGS)*. We next performed a technical and biological comparison in 41 patients for which there was ULP-WGS of cfDNA and WES of the matched metastatic tumor biopsy (Supplementary Fig. 7). For 22 MBC patients, we also performed WGS sequencing of the biopsy sample to 1× coverage and compared these to the ULP-WGS (Supplementary Fig. 4). These analyses serve as a technical comparison of the normalized log_2_ copy ratios between the two sample types. In addition, this analysis will highlight the similarities and differences in the CNAs between cfDNA and the tumor biopsy.

For the biopsy WES log_2_ comparison, we assessed log_2_ copy ratio at each 1 Mb bin in the cfDNA analysis but excluded bins that did not overlap at least one 50 kb bin in the tumor biopsy sample. The tumor biopsy CNA status, which was generated by TITAN^17^, was defined as the mean log_2_ copy ratio across all 50 kb bins overlapping the 1 Mb bin of interest. For the biopsy WGS (1×) comparison, we assessed the log_2_ copy ratio at each 1 Mb bin in both cfDNA and biopsy samples. We assessed the similarity between each cfDNA sample and the matching tumor biopsy by computing the Spearman correlation. Because the tumor fraction estimate from cfDNA may differ from the biopsy, the linear regression fit of these data may have varying slopes.

For the biopsy WES CNA comparison, we assessed CNA status at each 1 Mb bin in the cfDNA analysis but excluded bins that did not contain at least one SNP overlapping a WES target interval from the TITAN analysis. The CNA status at each SNP predicted by TITAN in the tumor biopsy served as the ground truth. To generate this ground truth data, each 1 Mb bin was assigned the TITAN CNA status of the overlapping SNP; for bins with more than 1 overlapping SNP, the most frequent CNA call was used. For the biopsy WGS (1×) comparison, we used ichorCNA to generate the CNA truth set for each 1 Mb bin from the WGS (1×) data and compared these to the CNA status at each 1 Mb bin in the cfDNA (ULP-WGS). The biopsy WGS (1×) and WES comparisons are presented in Supplementary Fig. 5c, d, respectively.

*Comparison of cfDNA tumor fractions between ULP and WES*. We compared the cfDNA tumor fraction estimates from ULP-WGS with the WES of the same matching sample analyzed by more established tools, ABSOLUTE and TITAN (Supplementary Fig. 15). Estimates of tumor content (i.e., tumor purity) and tumor cellular prevalence (i.e., cancer cellular fraction) from WES are estimated from deeper coverage data, which provides allelic information, using these more established approaches. Because the cfDNA sample may contain multiple subclones, the ULP tumor fraction may be an underestimate compared to the tumor content as estimated in WES. Ploidy estimation from ULP-WGS is less informed because of the absence of allelic information from low-coverage data. Discordant ploidy estimates from ULP-WGS when compared to WES leads to an underestimation of tumor fraction. Therefore, we identified samples with ploidy discordance as having differences >0.75. The Pearson correlation was then computed between ULP-WGS and WES across all cfDNA samples from concordant and discordant ploidy, separately (Supplementary Data 6).

### Theoretical power to determine WES coverage using ULP-WGS

In order to calibrate the amount of coverage for WES of cfDNA, one can determine the required sequencing depth based on theoretical power estimates computed using the tumor fraction estimation (Supplementary Fig. 25). This computation requires the ULP-WGS estimate of tumor fraction *α*, SNV multiplicity *M* (i.e., the number of chromosomes containing the variant), and the reference bias skewing of the sequencing library *w*. Let *p* be the expected allelic fraction of observing a heterozygous clonal (CCF = 1) SSNV. Let *M* = 1 by assuming a tumor ploidy of 2. Higher ploidy will increase the likelihood of observing the variant if the variant allele is amplified. The reference bias skew can be set *w* = 1, which assumes that there is skew and the variant allele is evenly represented relative to the reference allele.

$$p = \frac{{\alpha Mw}}{{\alpha \phi + 2\left( {1 - \alpha } \right)}}$$

To compute the theoretical power, we use the binomial test for observing 3 or more variant reads based on a given coverage *N* for the locus,

$$p\left( {X \ge 3} \right) = 1 - \left[ {{\rm{Bin}}\left( {0,N,p} \right) + {\rm{Bin}}\left( {1,N,p} \right) + {\rm{Bin}}\left( {2,N,p} \right)} \right]$$

### Mutation calling and filtering in whole-exome sequencing data

Illumina output was analyzed by the Broad Picard pipeline with bwa 0.5.9, resulting in BAM files aligned to hg19 with calibrated quality scores^46, 47^. To call somatic mutations in tumor biopsies and cfDNA, we used MuTect^11^. We used tools within the Firehose framework developed at the Broad Institute, which has been described previously^46, 48^. We assessed cross-sample contamination levels using ContEst^49^ and used these estimates as input for MuTect in order to set the lower bound of allele fraction accepted for mutation calling.

We called somatic SNVs using MuTect^11^ for cfDNA and tumor biopsy samples. Subsequently we filtered out potential artifactual OxoG mutations using the OxoG3 filter^50^ (https://www.broadinstitute.org/cancer/cga/dtoxog) and annotated mutation consequences with Oncotator^51^. We realigned reads around mutated sites with Novoalign (www.novocraft.com/products/novoalign/) to hg19 including decoy sequences and re-ran MuTect in order to filter out mutations in problematic regions. Finally, we filtered out SNVs using two panels of normal samples, the first with 8334 normal samples sequenced using Agilent exome capture, and the second with 140 normal samples using Illumina capture in order to filter out potential germline sites or recurrent artifactual sites. In both cases, we filtered out mutations called in 0.5% or more of the panel of normals samples that were seen in at least five samples. Additionally, we removed sites where at least 20% of samples across the panel of normals had reads supporting the mutation. Finally, we required that the site be covered by eight or more reads in at least 50% of samples and that, at most, 30% of samples had less than eight reads covering the site. For cfDNA samples, we applied an additional filter described below.

To call somatic insertions and deletion (indels), we used Strelka^52^ and annotated the mutation consequences using Oncotator^51^. To filter out potential false positives, we filtered indels against panels of normal samples, as described above for SNVs.

### cfDNA-specific mutation filtering in whole-exome sequencing data

Previous work has identified potential 8-oxoguanine artifacts in cfDNA at C>A bases^7^. This previous study identified a reference bias with G>T substitutions being more frequent than C>A substitutions, which suggests that oxidative damage is occurring during hybrid capture, as capture targets are designed with respect to the reference genome. We observe a clear reference bias in low allele fraction C>A mutations in cfDNA, whereas in the tumor biopsies, we do not observe a reference bias (Supplementary Fig. 26). Additionally, we observe a reference bias with more C>A than G>T mutations, which may be due to a difference in the reference genome strand used for capture bait design. This reference bias artifact is also distinct from previously identified 8-oxoguanine artifacts in exome sequencing data^50^. While there is a clear reference bias for low allele fraction mutations, the bias does not appear to be as strong for mutations at allele fraction >0.1. In order to filter out the potentially problematic sites, we decided to raise the tumor LOD score threshold^11^ LOD_*T*_ from 6.3 for C>A mutations at reference C bases. In order to choose an appropriate threshold, we evaluated the reference bias for C>A mutations in the 41 tumor biopsy samples and we raised the LOD_*T*_ threshold for C>A mutations at reference C bases in cfDNA until the reference bias for C>A mutations in the 59 cfDNA samples was equal to the reference bias for C>A mutations in the 41 tumor biopsies. This corresponded to a threshold of LOD_*T*_ > 11.72 for C>A mutations at reference C sites. We applied this filter to all cfDNA samples, and the mutational contexts following filtering are displayed for cfDNA and tumor biopsies in Supplementary Fig. 27.

We performed SSNV and indel analysis of cfDNA from 12 healthy donors to determine the false positive rate in our somatic mutation-calling pipeline. We applied the pipeline to WES of each healthy donor cfDNA, paired with its matched germline DNA. We calculated the false positive rate in each sample by dividing the number of non-synonymous SSNVs called by the total number of bases eligible for mutation calling, based on the coverage in the tumor and normal (i.e., sites with at least 14 reads in the cfDNA sample and 8 reads in the germline sample).

We also analyzed SCNA in the cfDNA of healthy donors using TITAN. The false positive rate was estimated as the percent of the genome altered: the total length of predicted SCNA segments per total genome length (3 × 10^9^).

### Copy number alteration analysis in whole-exome sequencing data

Both TITAN and ABSOLUTE tools were used to perform copy number analysis for cfDNA and tumor biopsy samples. We used results from both methods to help gain better confidence in the solutions produced. We found that the estimated global parameters of tumor purity and ploidy were very consistent between both approaches. Therefore, we decided to use ABSOLUTE results for most downstream analyses, but also used TITAN results for comparisons, particularly to ULP-WGS.

### Analysis using TITAN

We used the same pipeline described previously^17^ for TITAN. Briefly, the steps are as follows:

1. Identify heterozygous SNPs from the matched germline blood normal sample using Samtools *mpileup*. The set of *T* number of SNPs ${S_{1:T}}$ contained within HapMap3.37 variants were retained.
2. The reference *a*_*t*_ and non-reference *b*_*t*_ read counts at each site $t \in S$ were extracted from the tumor biopsy or cancer patient cfDNA sample. Chromosome X in male patients were excluded.
3. Read counts were computed at 50 kb bins using the HMMcopy Suite^43^. Centromeres are filtered based on chromosome gap coordinates obtained from UCSC for hg19, including bins that are 100 kb flanking up- and downstream of the gap. For WES analysis, only 50 kb bins overlapping the Illumina exome bait set intervals were retained.
4. The read coverage at 50 kb bins across the genome was corrected for GC-content and mappability biases independently for tumor/cfDNA and germline samples. The normalization approach is the same as described for ULP-WGS. Chromosome X was included for male patients; this is the only stage at which copy number is analyzed for chrX in males as allelic copy number was not performed on this chromosome. To compute the log2 copy ratios *l*_*t*_ at bin *t*, we first used the corrected read coverage of the matched germline normal *g*_*t*_ to normalize the corrected tumor/cfDNA coverage *r*_*t*_,
  $${l_t} = {\rm{log}}2\left( {\frac{{{r_t}}}{{{g_t}}}} \right)$$
  For ten healthy donor cfDNA samples with WES, we corrected for GC-content/mappability biases and computed the median at each bin across the five samples to generate a reference data set *h*_*t*_. Then, we further normalized the coverage using this reference, ${\hat l_t} = {l_t} - {\rm{log}}2\left( {{h_t}} \right)$
5. The data ${l_{1:T}}$, ${a_{1:T}}$, ${b_{1:T}}$ is input into the TitanCNA R package v1.10.1. Solutions were generated for 1 to 3 number of clonal clusters and ploidy initializations for 2 to 4. Optimal solutions were first selected by determining the optimal ploidy initialization. This was done by finding the consistently larger log-likelihood between the different ploidy initializations when comparing the solutions with the same number of clonal clusters. Then, when the optimal ploidy initialization is determined, the solution with the optimal number of clonal cluster is selected using the minimum *S_Dbw* validity index (using both log ratio and allele ratio). Additional comparisons of purity and ploidy estimates with ABSOLUTE results led to re-selection of solutions for some samples. The specific arguments used in TitanCNA: maxCN = 8, alphaK = 1000, txn_exp_len = 1e15, txn_z_strength = 1, minDepth=10, maxDepth=1000. Default values were used for remaining arguments.
6. Output SCNA state definitions: HET—heterozygous diploid, two copies; DLOH—deletion LOH, one copy; NLOH—copy neutral LOH, two copies; GAIN—copy number gain, three copies; ALOH—amplified LOH; three or more copies, ASCNA—allele-specific copy number amplification; four or more copies; BCNA—balanced copy number amplification; four or eight copies; UBCNA—unbalanced copy number amplification; five or more copies.

The parameters for the optimal solutions are listed in Supplementary Data 6 and the segments are found in Supplementary Data 4. The TitanCNA package was obtained from https://github.com/gavinha/TitanCNA.

### Analysis using ABSOLUTE

To evaluate SNVs in paired samples, we needed to consider the union of mutations called in the two samples. In order to evaluate the sites that were not initially called, we used forced calling to quantify the number of alternate reads at each mutant site. We considered reads that were not duplicates, had a recalibrated base score at the mutant site ≥20 and had a mapping quality ≥5, and calculated the number of alternate and reference reads. For tumor biopsy to cfDNA comparisons, we used forced calling for the union of mutations called in the tumor and cfDNA. Separately, for the evaluation of patients with two cfDNA samples, we used forced calling for the union of mutations called in the two cfDNA samples (not including mutations called in the tumor biopsy). We then filtered out noncoding (e.g., intronic or UTR sites) and used these force called mutations in paired samples as input for ABSOLUTE.

To estimate somatic copy number from WES, we used ReCapSeg (http://gatkforums.broadinstitute.org/categories/recapseg-documentation), which calculates proportional coverage for each target region (i.e., reads in the target/total reads) and then normalizes each segment using the median proportional coverage in a panel of normal samples. Then, the sample is projected to a hyperplane defined by the panel of normals in order to reduce noise and estimate the tumor copy-ratio. For tumor biopsies, we used a panel of normals samples sequenced with the same capture technology used to sequence the tumor (i.e., Agilent or Illumina capture). For cfDNA samples, we used a panel of normals that also included the healthy donor cfDNA samples in order to reduce noise specific to cfDNA samples. WES copy-ratio profiles were then segmented with CBS^53, 54^. To estimate allelic copy number, we called germline heterozygous sites in the germline normal sample using GATK Haplotype Caller^47, 55^ and then evaluated the reference and alternate read counts at the germline heterozygous sites in order to assess the contribution of each homologous chromosome. Finally, we segmented the allele specific copy ratios using PSCBS^54^ and used the resulting copy ratios (and the force called SNVs and indels) as input for ABSOLUTE ^16, 56^ to estimate the sample purity and ploidy and estimate absolute allelic copy number as well as the CCF of SCNAs and SNVs. We filtered out recurrent artifactual segments that overlapped the centromere of chromosome 1 before input to ABSOLUTE. ABSOLUTE solutions were manually reviewed, and we selected purity/ploidy solutions. As we expected that cfDNA would be derived from tumor cells related to those in the tumor biopsies, we expected that the ploidy of tumor biopsies and cfDNA samples would be consistent. Thus, we selected solutions that maintained consistent genome doubling status between cfDNA and tumor biopsies. Since we did not estimate allelic copy number for the X chromosome, and we did not assess the clonality of mutation on the X chromosome with the exception of three *AR* hotspot mutations for which we manually assigned the mutations a CCF of 1 based on their allele fractions (Supplementary Fig. 22).

### Copy number alteration and gene overlap analysis

For both ULP-WGS and WES SCNA analysis, gene-level alterations were determined using the list of 19,378 known coding genes from GENCODE^57^ v19. The copy number status was assigned based on the largest overlap with the predicted SCNA segment from the algorithms.

### Comparison between WES of cfDNA and metastatic tumor biopsy

First, we considered the clonal and subclonal coding SSNVs that had initially been detected (i.e., called by MuTect and passing MuTect filters) in the tumor biopsy. We then evaluated the loci of these mutations in the matched cfDNA sample to look for any evidence of the mutation in cfDNA. We also considered the mutations initially detected in the cfDNA sample and assessed their detection in the tumor. We also assessed the overlap of cfDNA mutations that were also detected in a later cfDNA time point. We used cfDNA-exclusive mutations (i.e., mutations that were detected in cfDNA and powered but not confirmed in tumor biopsies), and evaluated the loci of these mutations in the cfDNA sample taken at a later time point (*t*_*2*_). Details of the comparison are described next.

First, we considered the clonal and subclonal coding SSNVs that had initially been detected (i.e., called by MuTect and passing MuTect filters) in the tumor biopsy. We used the predicted ABSOLUTE CCF to assignments clonal (≥0.9 CCF) and subclonal (<0.9 CCF) in the tumor biopsy. We then evaluated the loci of these mutations in the matched cfDNA sample to look for any evidence of the mutation in cfDNA. For each locus,

1. If there were ≥3 reads supporting the mutant allele, then considered confirmed to be mutated in cfDNA. Let *c* be the set of confirmed mutations.
2. If a locus had <3 reads of the mutant allele in cfDNA, then it fell into one of two categories:

i) If the site had <0.9 power (based on power to observe ≥3 mutant allele reads for a mutation with CCF = 1 and multiplicity = 1; see section “Theoretical power estimation”), then we considered the mutation unpowered, and these were excluded in the overlap comparison.
ii) If the mutation had power ≥ 0.9 but had < 3 alternate reads, then the mutation was powered by not confirmed in cfDNA. Let *c*′ be the set of unconfirmed (powered) mutations.

Them we computed the overlap as ${\rm{fraction}}\,{\rm{overlap}} = \frac{c}{{c + c\prime }}$. The overlap was computed separately for clonal and subclonal tumor biopsy mutations.

Similar to the above approach, but swapping the two samples in the comparison, we used the mutations initially detected in the cfDNA sample and assessed their detection in the tumor. We performed the same power analysis as described above.

We assessed the overlap of cfDNA mutations that were also detected in a later cfDNA time point. We used cfDNA-exclusive mutations (i.e., mutations that were detected in cfDNA and powered but not confirmed in tumor biopsies), and evaluated the loci of these mutations in the cfDNA sample taken at a later time point (*t*_2_). We assessed whether these SSNVs were confirmed in *t*_2_, again using the same power calculation described above. The confirmed cfDNA-exclusive mutations are annotated in Fig. 2b in black. The overlap fraction was computed as described above.

### Analysis of clonal dynamics using PHYLOGIC

To assess mutation clonality in paired samples, we used PHYLOGIC^39, 58^ to perform clustering of ABSOLUTE CCFs, as described previously. For comparisons of tumor biopsies and cfDNA samples (Supplementary Fig. 19), we evaluated mutations force called using biopsies and cfDNA samples. For evaluation of clonal dynamics between multiple cfDNA samples from the same patient (Supplementary Fig. 21), we evaluated mutations force called using the two cfDNA samples (and not the tumor biopsy). In all cases, we used 2500 MCMC iterations with half discarded as burn-in and a negative binomial (*r* = 10, mu = 10) prior for the number of mutation clusters. For patient MBC_284 (Fig. 2), we assessed clonal shifts in pairs of samples using mutations force called in tumor biopsy and both cfDNA samples together, and we used a negative binomial (*r* = 16, mu = 16, 250 iterations) prior. In PHYLOGIC clustering with all pairs of samples for this patient, we discarded mutations that had zero supporting reads in both samples during clustering (e.g., if a mutation was called in the second cfDNA sample but had zero supporting reads in either the tumor biopsy or the first cfDNA sample). Additionally, when performing clustering analysis, we filtered out mutations from a list of sites determined to be problematic based on previous Phylogic analyses^39, 58^. The software used for this analysis can be obtained from http://www.broadinstitute.org/cancer/cga/acsbeta.

### Mutation significance analysis

We used MutSig2CV^23, 24^ to identify genes mutated significantly above the background rate. In order to compare results between cfDNA and tumor biopsies, we performed significance analysis separately for cfDNA WES samples and tumor biopsy samples for the 27 patients with MBC and the 14 patients with metastatic prostate cancer. As there is limited power to discover novel genes in such small cohorts, we focused on comparing the genes identified by the cfDNA and tumor biopsy significance analyses, and we used a *q* < 0.1 threshold. We report MutSig2CV q values in Supplementary Data 8.

To assess whether any of the significantly mutated genes were enriched for non-synonymous mutations in the 20 ER+/Her2− MBC cases as compared to primary ER+/Her2− breast cancer, we compared these samples to tumors the from TCGA breast cancer cohort^25^. We used the TCGA mutational data (http://cbio.mskcc.org/cancergenomics/tcga/brca_tcga) from 279 ER+, Her2−, non-metastatic cancers. We only considered non-synonymous mutations for this analysis. We evaluated the enrichment of each of the four significantly mutated genes in the metastatic cases as compared to the non-metastatic cases in TCGA using Fisher’s exact test and we performed a Bonferroni correction within the cfDNA vs. TCGA and tumor biopsy vs. TCGA analysis.

### Mutation signature analysis

We used a previously described Bayesian NMF framework to discover mutational signatures in cfDNA and tumor biopsies^29, 59, 60^. For the purposes of mutation signature analysis, we excluded the tumor biopsy and two cfDNA time points from patient CRPC_468 which had an extremely high mutation rate and displayed evidence for microsatellite instability (MSI). Both the tumor biopsy and cfDNA supported a homozygous deletion of *MSH2*, which is the likely cause of the MSI signature present in these samples. We performed mutational signature discovery using all called coding mutations (annotated with their tri-nucleotide sequence context) in the remaining 40 tumor biopsy samples, the matched 40 cfDNA samples as well as the 17 cfDNA samples taken at later time points. In order to identify the number of mutational signatures *k* and their activity in all samples, we used 50 iterations starting with random initial conditions. We found that 45 iterations converged to *k* = 3 and five iterations converged to *k* = 4, so we selected the solution with *k* = 3 that had the maximum posterior probability. We compared the three discovered signatures to COSMIC signatures^30, 61^ using cosine similarity. We then assigned each mutation to the signature with the maximum probability of association^59^ and calculated the fraction of mutations assigned to each of the three signatures in every sample. To assess enrichment of the homologous recombination deficiency-associated signature in samples with BRCA-deficiency, we compared the signature fraction between samples with homozygous *BRCA1* or *BRCA2* alteration (through a combination of germline loss of function mutation and somatic LOH or somatic homozygous deletion) and those samples without homozygous *BRCA1/2* alterations.

### Neoantigen analysis

In order to compare neoantigen prediction between cfDNA and tumor biopsies, we first called germline MHC Class I alleles (*HLA *− *A*, *HLA *− *B*, *HLA *− *C*) from normal tumor exome sequencing data with Polysolver ^62^. We considered missense SNVs, and used NetMHCpan 2.4^35, 63^ to predict the binding affinity of all potential 9-mers overlapping the mutated peptide with respect to all six germline MHC Class I alleles. We considered a site a predicted neoantigen if it’s predicted IC50 was below 500 nM for any HLA allele.

### Data availability

Sequencing data have been deposited into dbGaP under accession code phs001417.v1.p1.

## Electronic supplementary material

Supplementary Information

Description of Additional Supplementary Files

Supplementary Data 1

Supplementary Data 2

Supplementary Data 3

Supplementary Data 4

Supplementary Data 5

Supplementary Data 6

Supplementary Data 7

Supplementary Data 8
