# Supplementary material for: Scalable whole-exome sequencing of cell-free DNA reveals high concordance with metastatic tumors
Source: Nat Commun. 2017 Nov 6;8:1324. doi: 10.1038/s41467-017-00965-y (PMC5673918; doi:10.1038/s41467-017-00965-y)
Supplement: Supplementary file 1 — Supplementary Information [file 41467_2017_965_MOESM1_ESM.pdf]

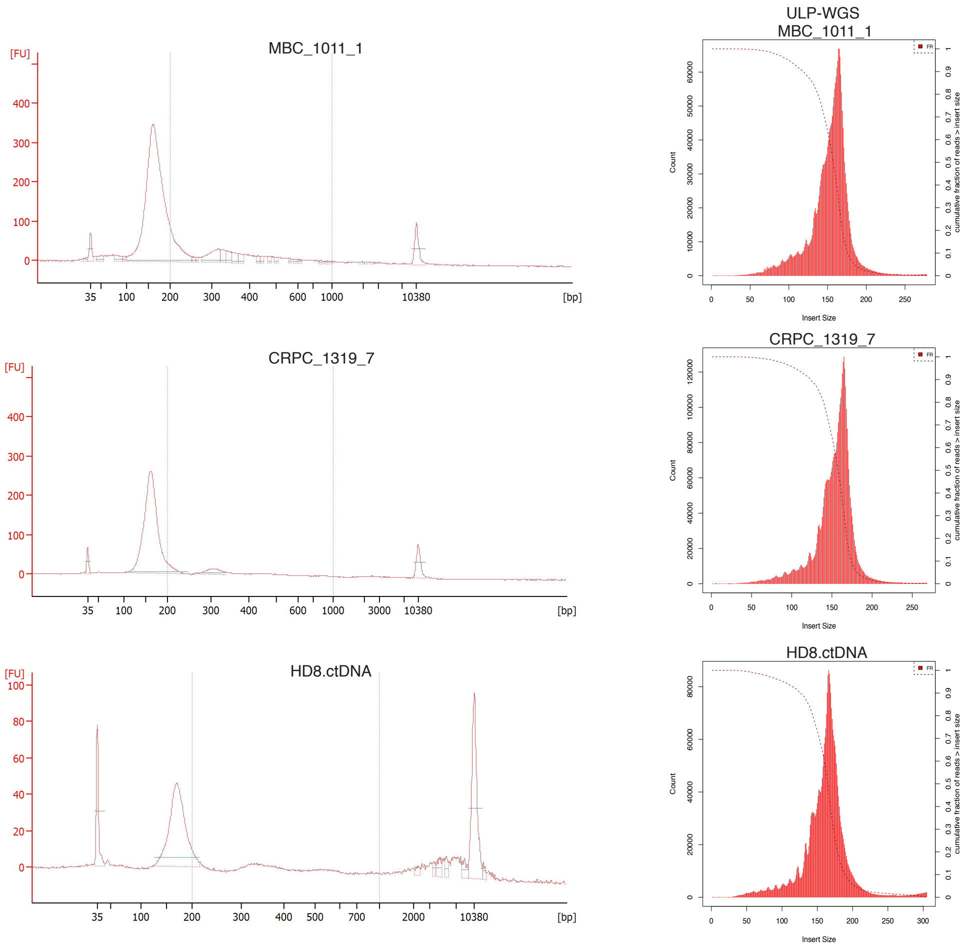

Supplementary Figure 1: BioAnalyzer and insert size distribution of cfDNA. (Left) Electropherogram showing the size distribution of cfDNA. Data was generated using a High Sensitivity DNA chip on the Agilent 2100 Bioanalyzer. Markers were used for 35bp and 10,380bp. (Right) Insert size histograms are all generated by Picard (version 1.1090) `CollectInsertSizeMetrics` for ULP-WGS using 5ng libraries. Examples from metastatic breast cancer (MBC), prostate cancer (CRPC) and a healthy donor (HD) individuals are shown.

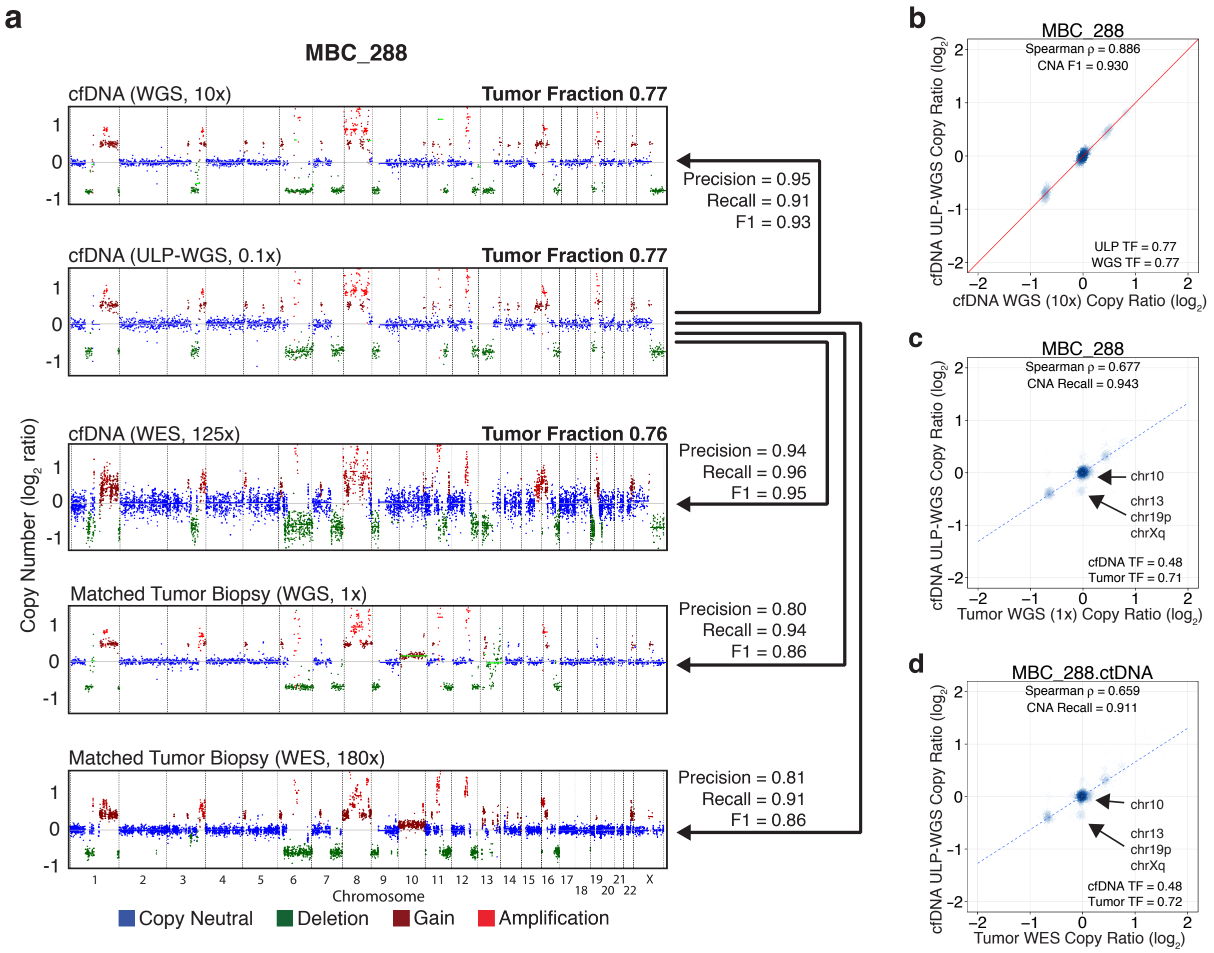

Supplementary Figure 2: Performance of ULP-WGS (0.1x) compared to the deeper sequencing of the same cfDNA sample (10x WGS and WES) and the matched tumor biopsy (1x WGS and WES) for patient MBC_288. a) Copy number profiles for cfDNA WGS (Titan, 1Mb bins), cfDNA ULP-WGS (1Mb bins) cfDNA WES (Titan, 50kb bins), matched tumor biopsy WGS, and matched tumor biopsy WES (Titan, 50kb bins). Performance was computed based on binary classification metrics of precision and recall (see Methods) for comparisons. Comparison of normalized copy ratios ($\log_2$) between cfDNA ULP-WGS and (b) deep WGS of the same cfDNA sample, (c) 1x WGS of the tumor biopsy, and (d) WES of the tumor biopsy are shown. Biological differences in copy number events in chromosome 10, 13, 19p, and Xq are indicated with arrows. Red line denotes y=x; dashed blue line denotes model fit using linear least squares regression.

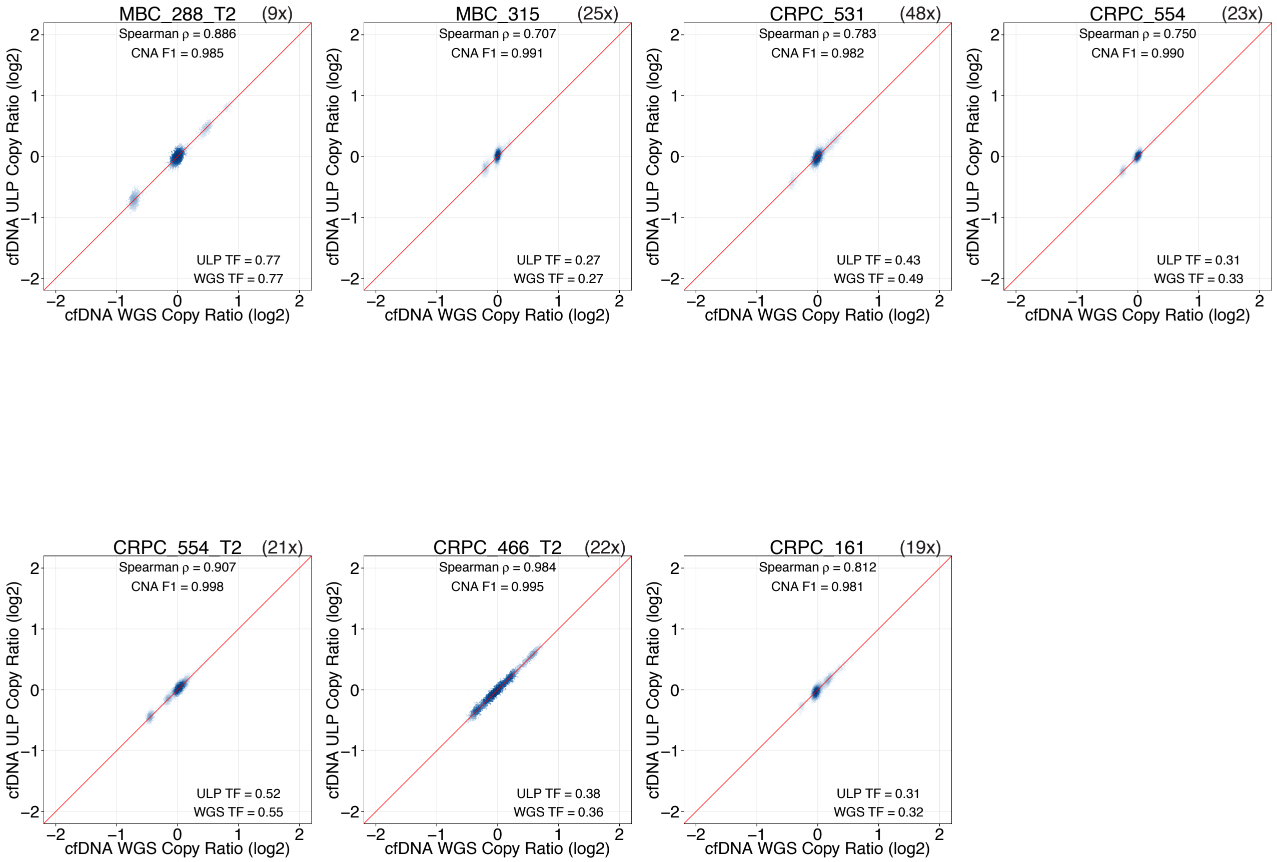

Supplementary Figure 3: Comparison between ULP-WGS and WGS (>10x) of cfDNA for the same 7 metastatic breast (MBC) and prostate (CRPC) cancer patients. $\log_2$ copy ratios were computed as normalized coverage (see Methods) for 1Mb bins. See Methods for description of comparison and Spearman correlation (ρ).

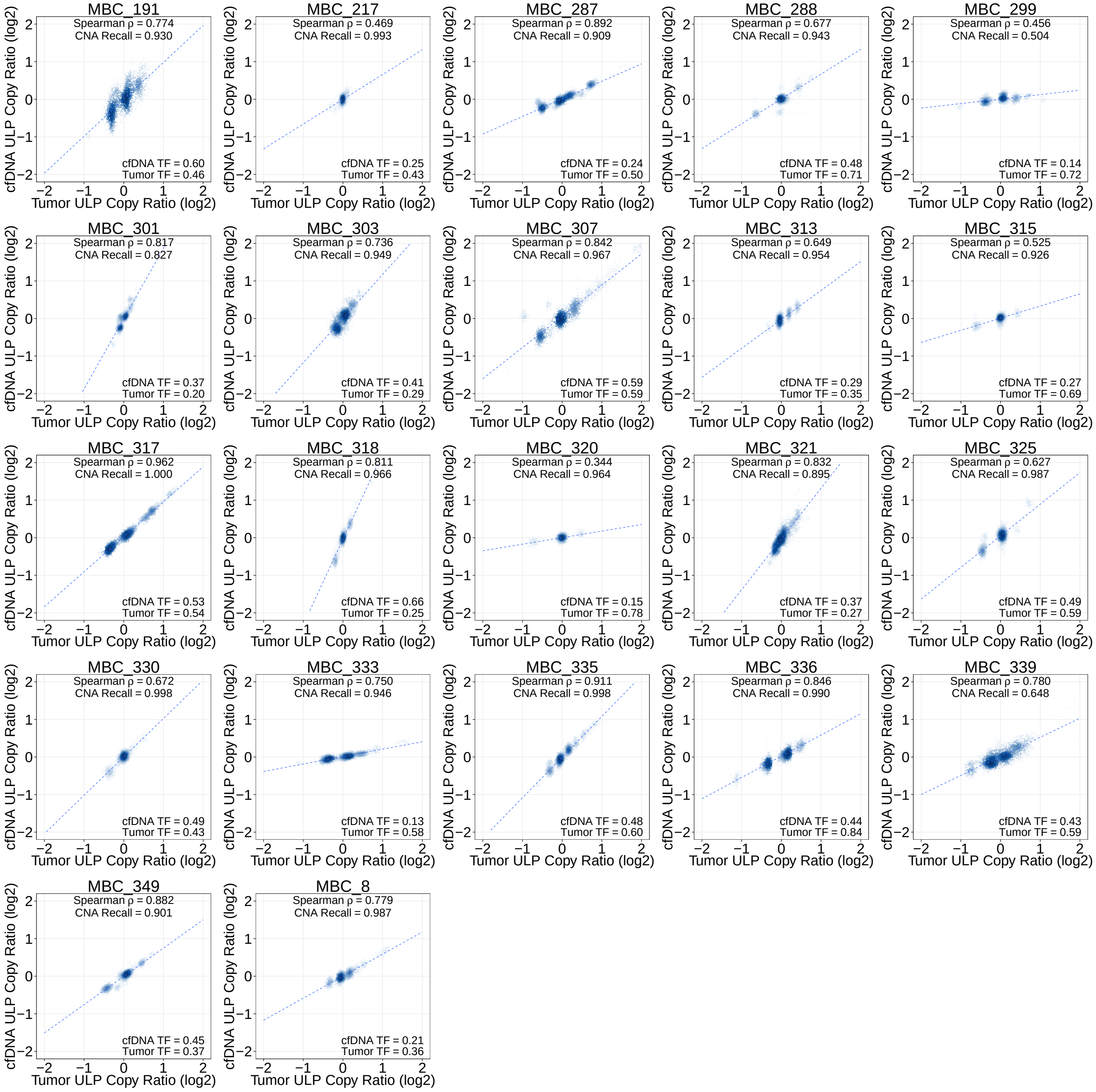

Supplementary Figure 4: Comparison between ULP-WGS of cfDNA and WGS (1x) of tumor biopsy for 22 metastatic breast (MBC) cancer patients. $\log_2$ copy ratios were computed as normalized coverage (see Methods) for 1Mb bins. See Methods for description of comparison and Spearman correlation (ρ).

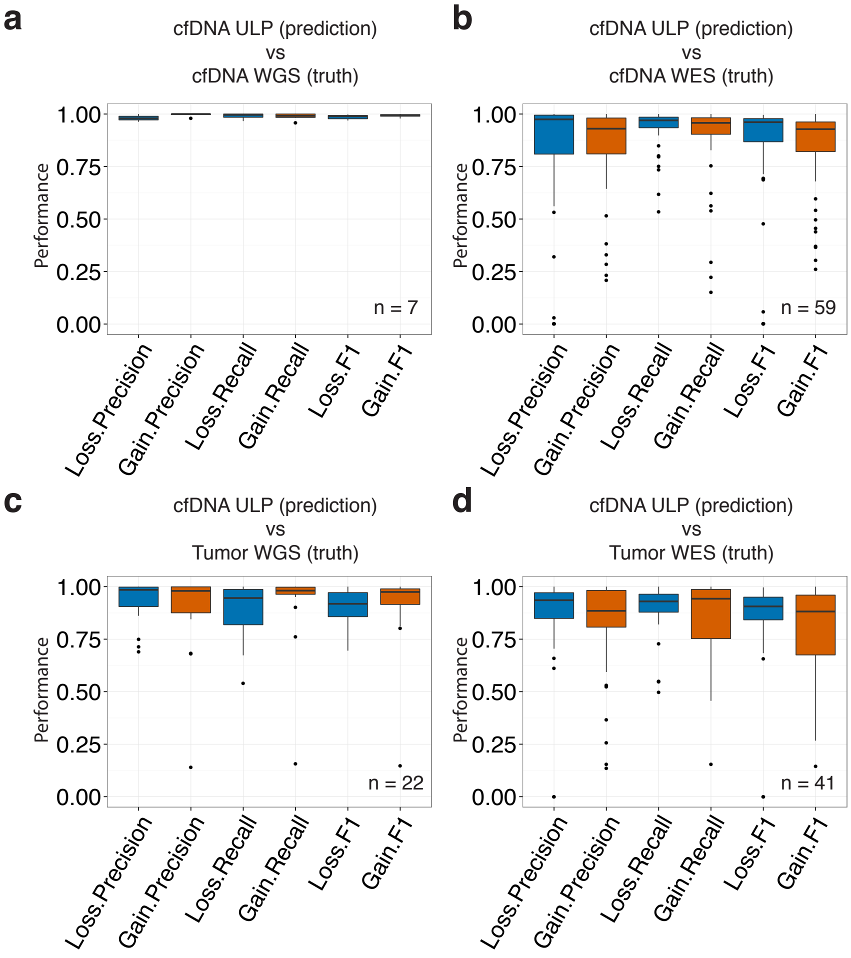

Supplementary Figure 5: Comparison of SCNA between ULP-WGS of cfDNA and (a) WGS (>10x) of cfDNA from the same plasma collection, (b) WES of cfDNA from the same collection, (c) WGS (1x) of tumor biopsies, and (d) WES of tumor biopsies. See Methods for details on how the comparisons were performed. The SCNA status for ULP-WGS at each 1Mb bin was compared to SCNA results from four datasets analyzed using Titan for (a, b, d) and IchorCNA for (c). See Methods for description of comparison and definition of precision, recall, and F1-score.

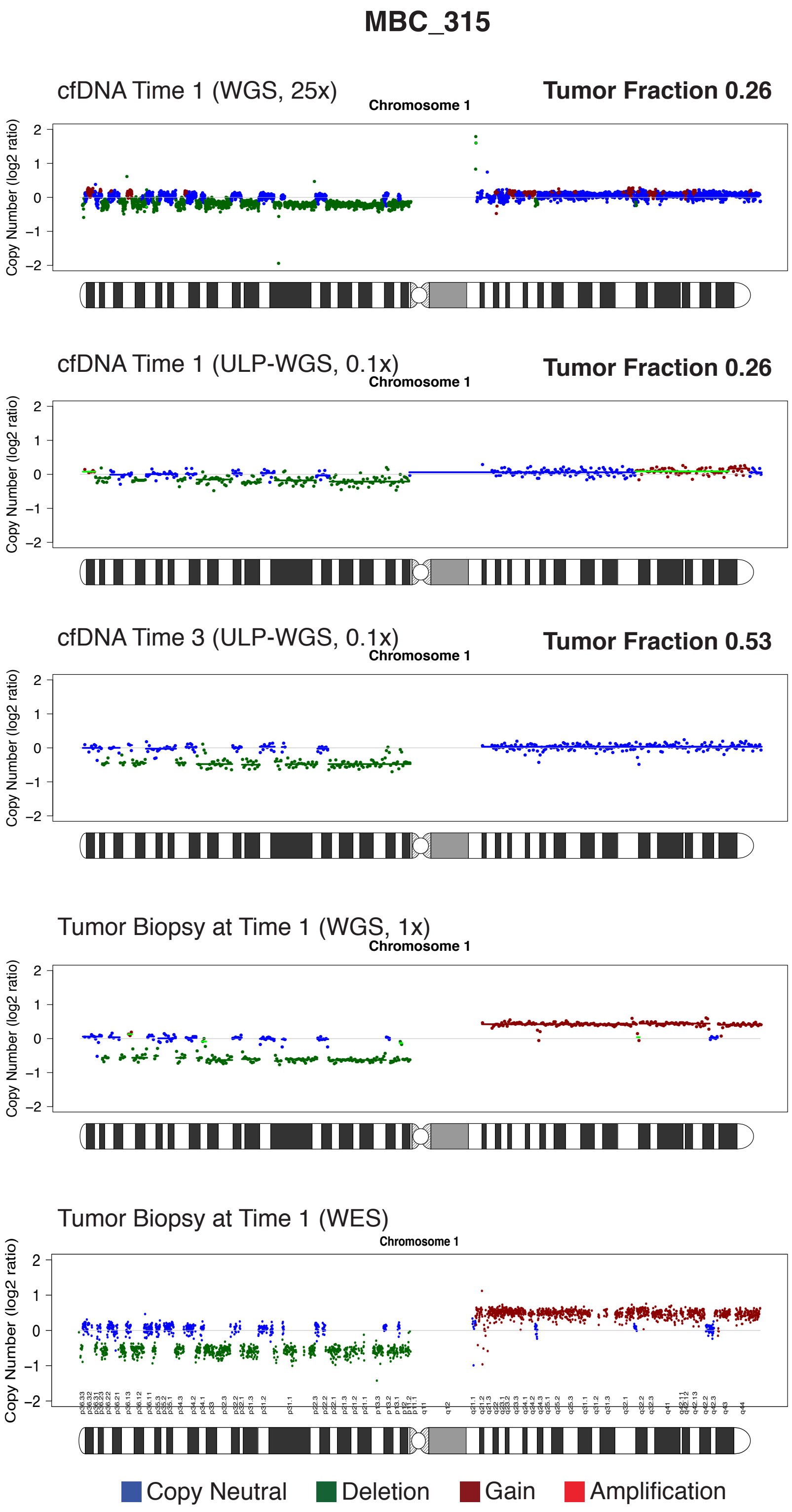

Supplementary Figure 6: Chromothripsis detected in cfDNA for metastatic breast cancer patient MBC_315 (chr1). Copy number profiles are shown for cfDNA WGS (Titan, 50kb bins), cfDNA ULP-WGS (500kb bins), and tumor biopsy WES (Titan, 50kb bins). Analysis of WGS and ULP-WGS was not done using a matched normal; WGS of a matched normal was not available. Analysis of WGS and WES data were performed using Titan, but only read coverage-based results (from Steps 1-4 Methods: Analysis using TITAN) are shown in the plots.

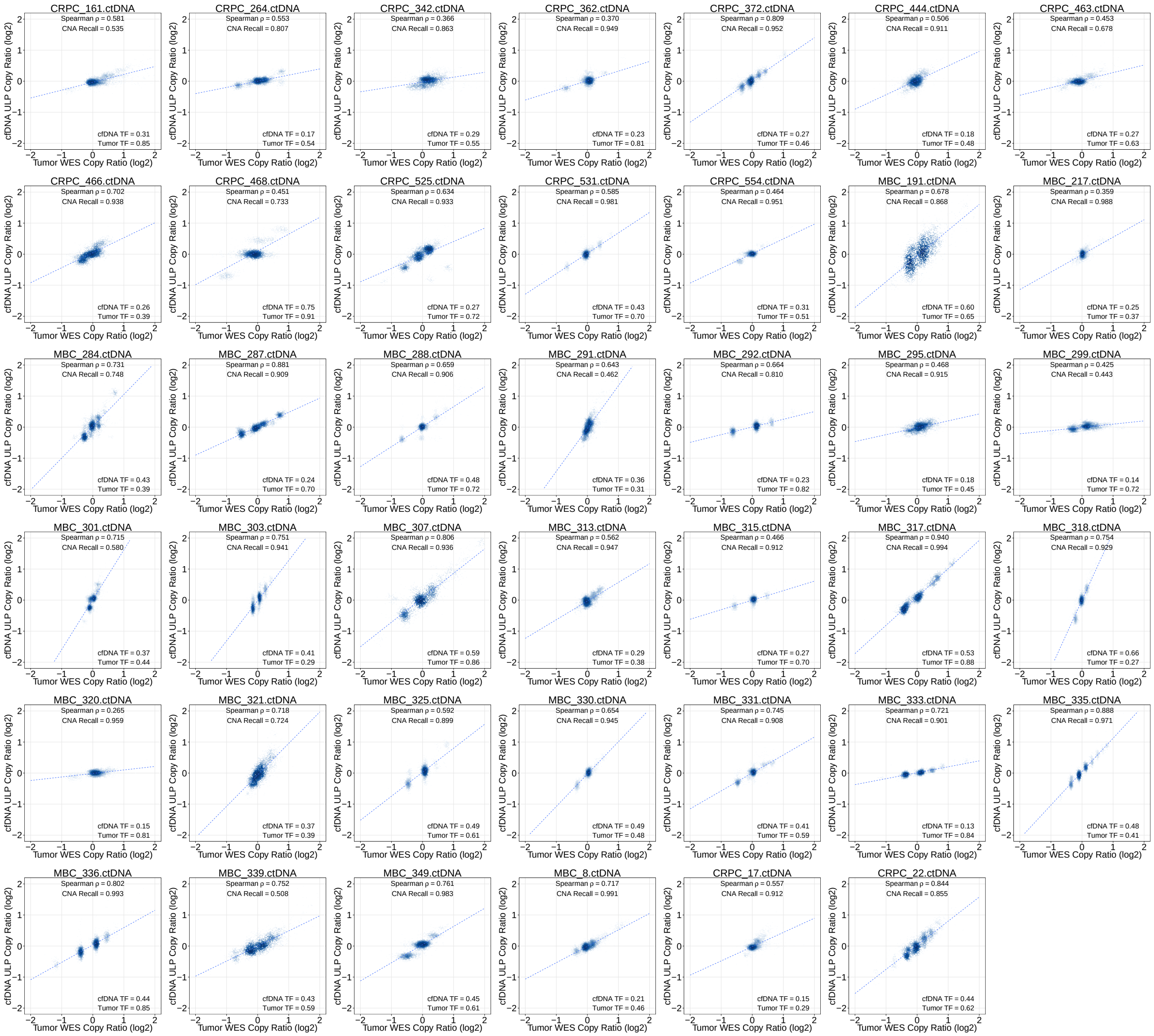

Supplementary Figure 7: Comparison between ULP-WGS of cfDNA and WES of tumor biopsy for 41 metastatic breast (MBC) and prostate (CRPC) cancer patients. $\log_2$ copy ratios were computed as normalized coverage for cfDNA ULP-WGS (1Mb bins) and tumor biopsy WES (Titan, 50kb bins). See methods for description of comparison and Spearman correlation (ρ).

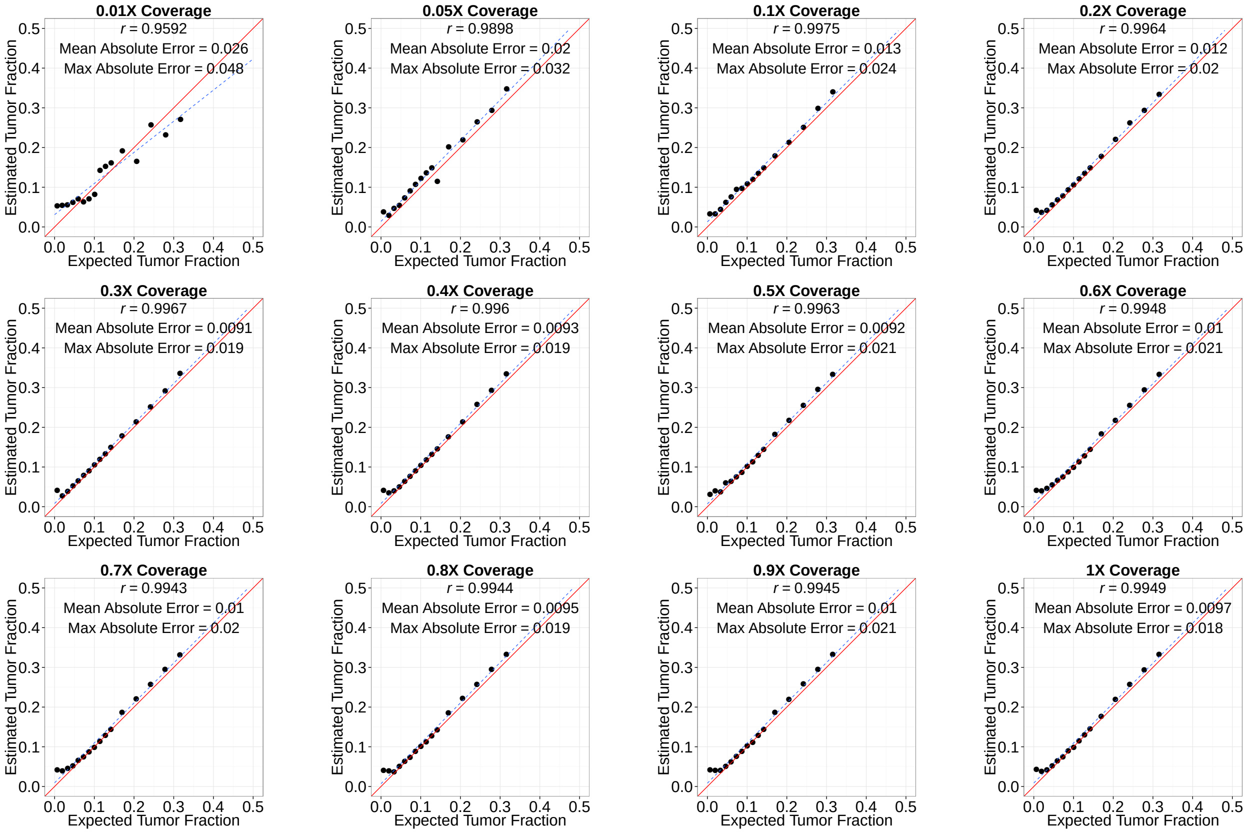

Supplementary Figure 8: Performance of ULP-WGS tumor fraction estimation in admixture experiment using MBC_288. The ULP-WGS algorithm was applied to estimate tumor fraction from admixture benchmark samples with expected (known) tumor fractions ranging 0.007-0.33 for each depth of sequencing coverage 0.01x-1x (Methods). Correlation was computed using Pearson's r; mean and max absolute error was computed across 16 admixture samples for each coverage. Red line denotes $y = x$; dashed blue line denotes model fit using linear least squares regression.

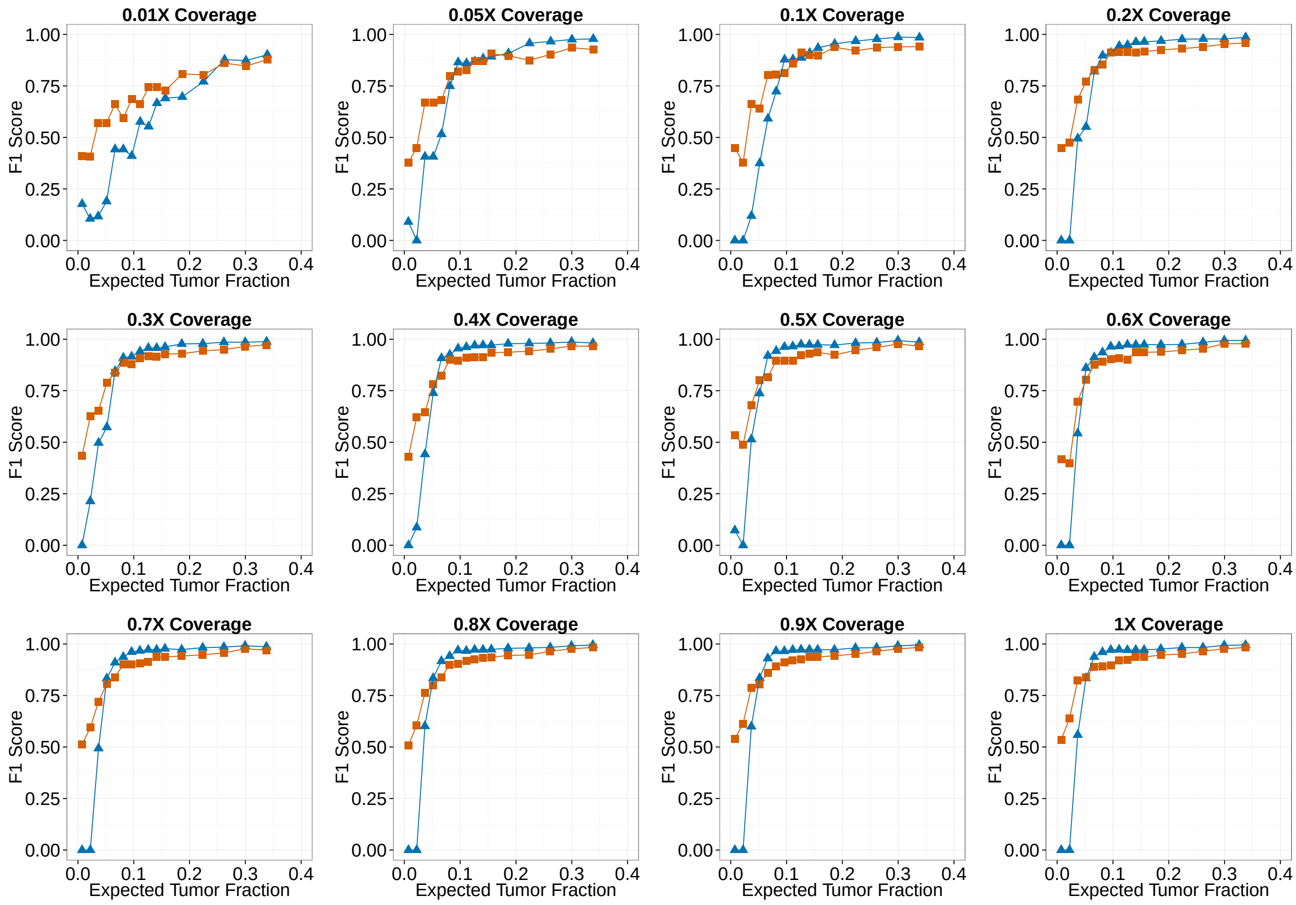

Supplementary Figure 9: Performance (F-measure) of ULP-WGS CNA predictions in admixture experiment using MBC_288. The ULP-WGS algorithm was applied to predict SCNA deletions (blue triangles) and gains (red squares) from admixture benchmark samples with expected (known) tumor fractions ranging 0.007-0.33 for each depth of sequencing coverage 0.01x-1x and ground truth SCNA. See Methods for definitions of performance metric.

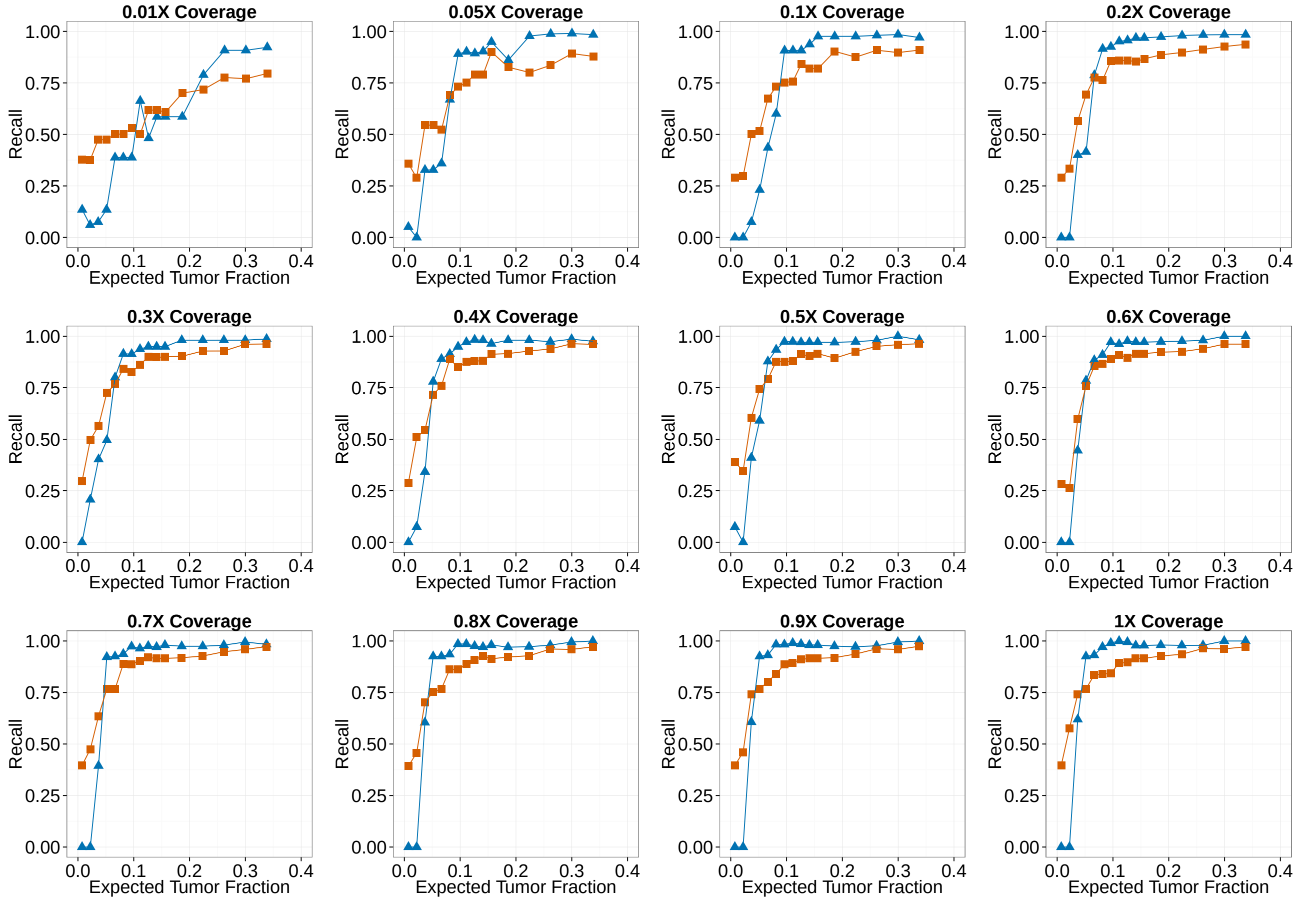

Supplementary Figure 10: Performance (recall/sensitivity) of ULP-WGS CNA predictions in admixture experiment using MBC_288. The ULP-WGS algorithm was applied to predict SCNA deletions (blue triangles) and gains (red squares) from admixture benchmark samples with expected (known) tumor fractions ranging 0.007-0.33 for each depth of sequencing coverage 0.01x-1x and ground truth SCNA. See Methods for definitions of performance metric.

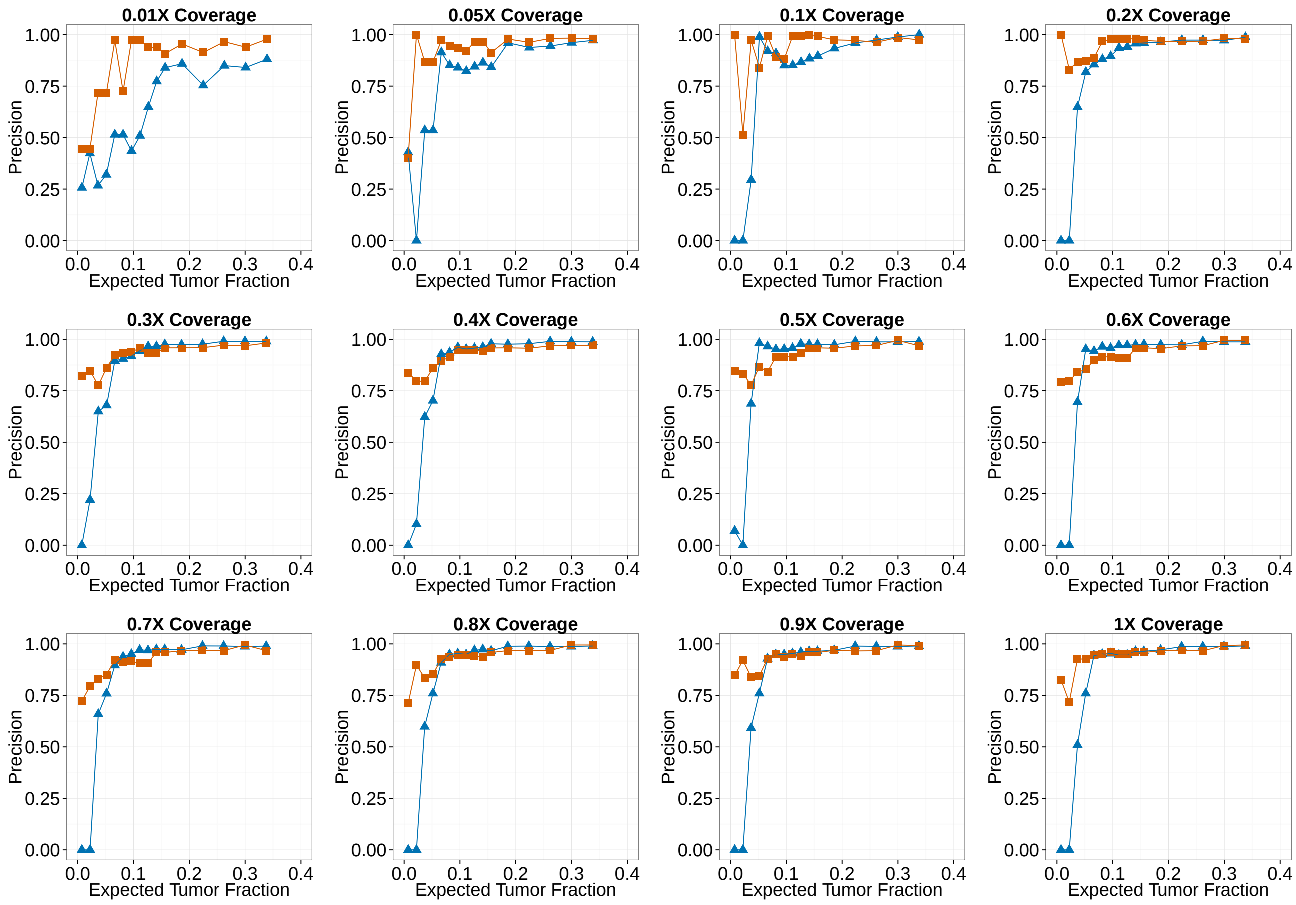

Supplementary Figure 11: Performance (precision) of ULP-WGS CNA predictions in admixture experiment using MBC_288. The ULP-WGS algorithm was applied to predict SCNA deletions (blue triangles) and gains (red squares) from admixture benchmark samples with expected (known) tumor fractions ranging 0.007-0.33 for each depth of sequencing coverage 0.01x-1x and ground truth SCNA. See Methods for definitions of performance metric.

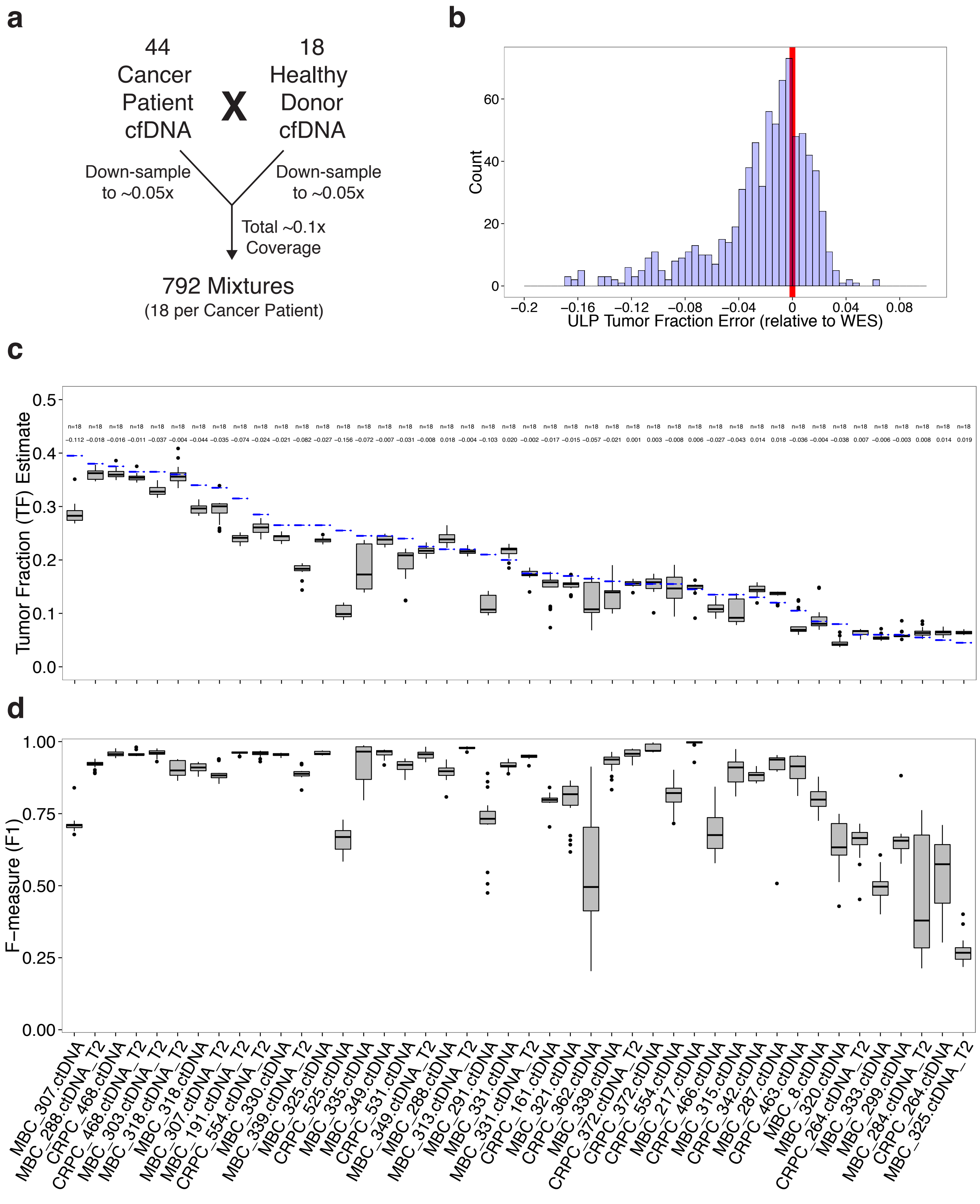

Supplementary Figure 12: Mixture simulation analysis using 44 cancer patient cfDNA samples and 18 healthy donors. (a) Each cancer patient and healthy donor sample was downsampled to ∼0.05x and mixed together to generate 792 total mixtures of ∼0.1x coverage. (b) The tumor fraction error was computed as the difference between the prediction from the mixture and the estimated tumor purity from the matching WES sample. The error for all mixtures are shown. (c) The tumor fraction estimates for each cancer patient sample (n=18 mixtures) are shown with the boxplots indicating the variance in the estimates (1.5*IQR). The median error (deviation) relative to the expected tumor fraction are indicated. (d) The F-measure CNA performance as compared to the truth CNA calls from WES are shown. See Methods for description of the mixture procedure.

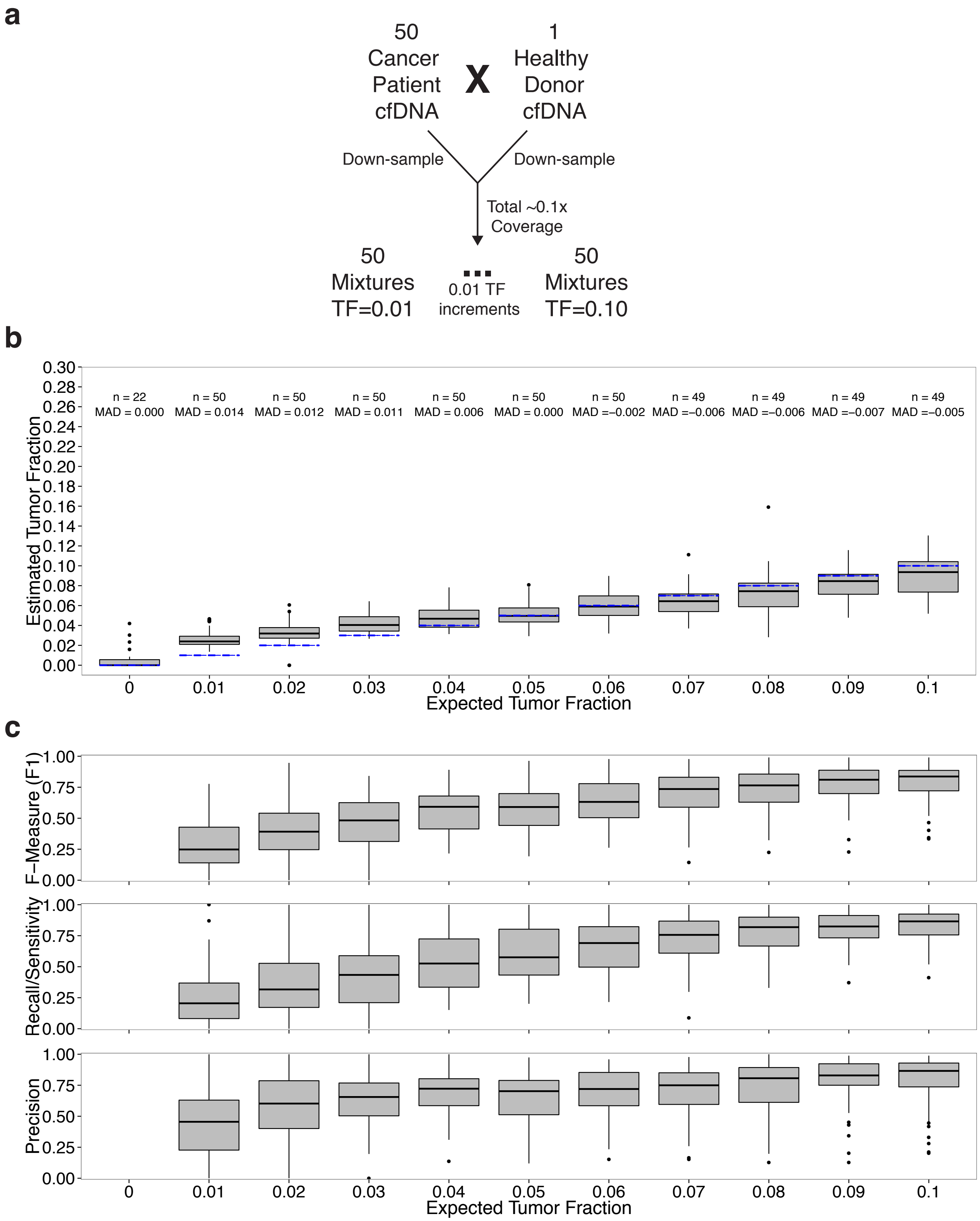

Supplementary Figure 13: Exact tumor fraction mixture simulation analysis using 50 cancer patient cfDNA samples. (a) For each cancer patient, the specific number of reads giving an exact tumor fraction (0.01 to 0.1) are mixed with a healthy donor (HD_2). (b) The tumor fraction estimates for each expected tumor fraction (n>49) are shown as boxplots. The median absolute deviation of the error relative to the expected tumor fraction are indicated. (c) The F-measure, recall, and precision for the CNA performance as compared to the truth CNA calls from WES are shown. See Methods for description of the mixture procedure.

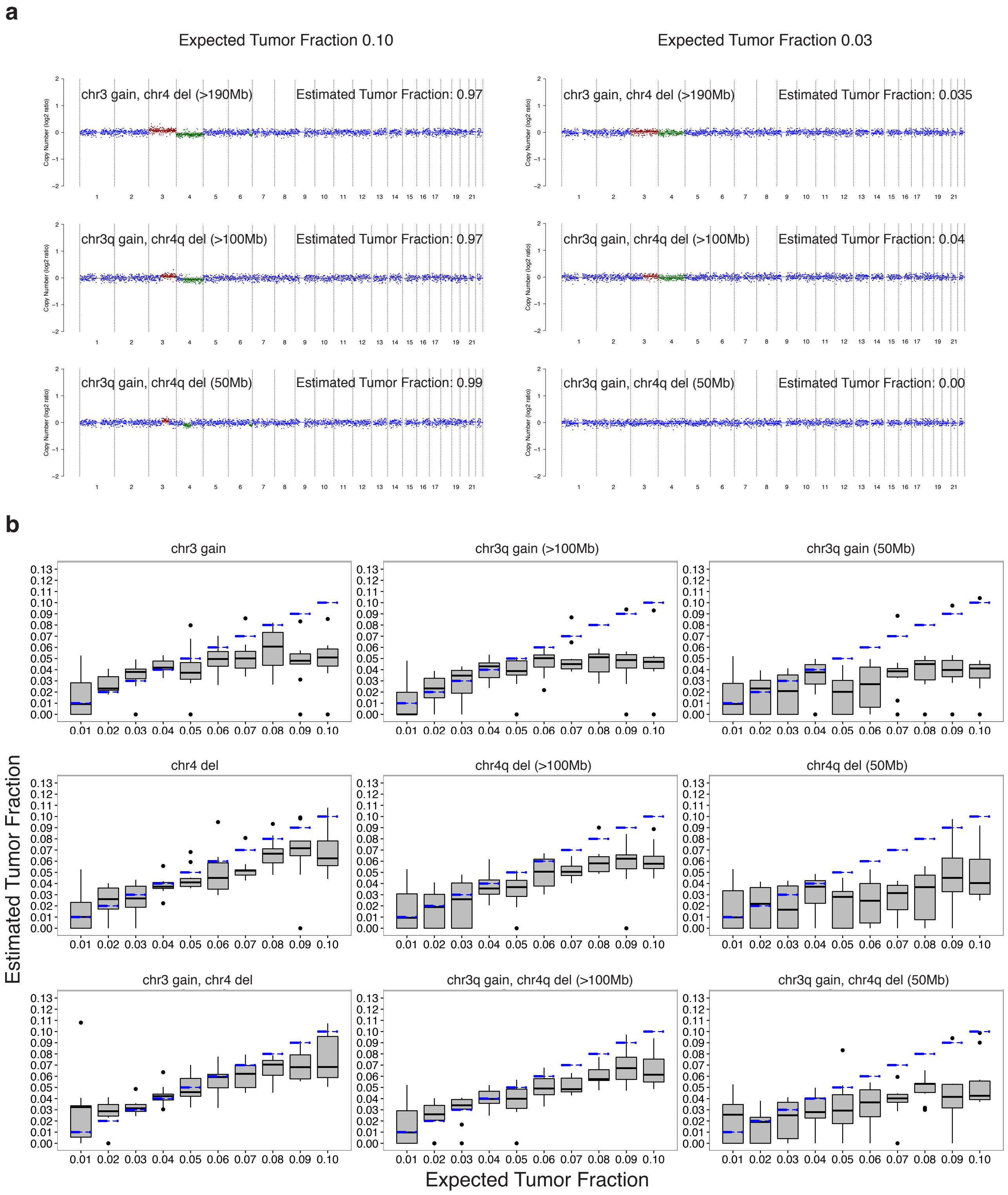

Supplementary Figure 14: Spike-in mixture simulation to assess the minimum number, size, and magnitude of CNA event required to detect tumor-derived DNA and to estimate tumor fraction. (a) Examples of small (50Mb), arm-level (>100Mb), and whole-chromosome level CNA loss in chr4 and gain in chr5 shown for expected tumor fractions of 0.10 and 0.03. Deletion, gain, and copy neutral segments are indicated in green, red, and blue, respectively. (b) Estimated tumor fraction estimates for 900 spike-in mixture simulation samples for a single gain, a single loss, and 1 gain plus 1 loss for the three event sizes across expected fractions of 0.01 to 0.10. the performance for estimating tumor fraction is high for the scenarios: (1) an arm loss and an arm gain are both present and (2) either a whole chromosome gain or a whole chromosome loss. For detecting presence of tumor at a limit-of-detection of 0.03 tumor fraction, a single arm loss or a single arm gain was sufficient. See Methods for description of the mixture procedure.

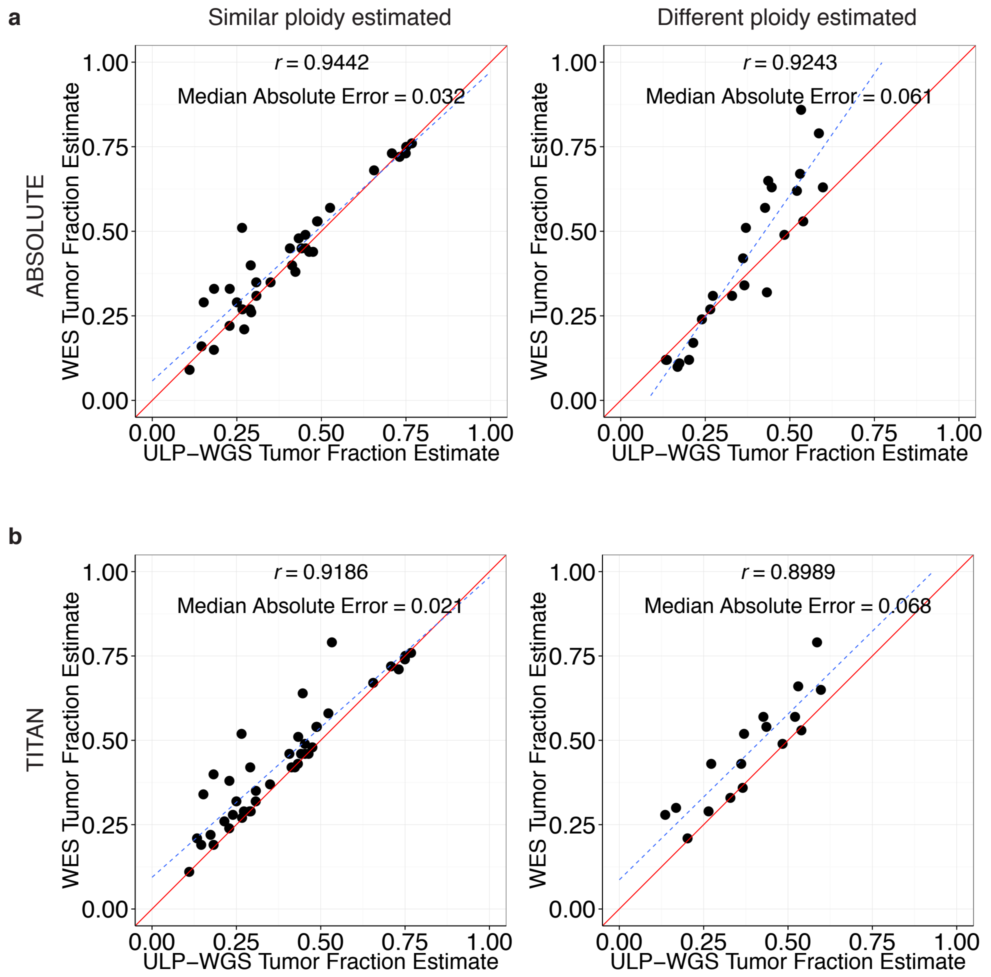

Supplementary Figure 15: Comparison of tumor fraction estimates between ULP-WGS and WES of the same cfDNA collection analyzed using Absolute (a) and Titan (b) for 59 samples from 41 patients (Supplementary Data 6). The comparison is divided into samples for which the tumor ploidy estimates for both sample types were similar (left; difference ≤ 0.75) or different (right; difference > 0.75 and ploidy ≤ 1.5). See Methods for details on the comparison of tumor fraction estimates.

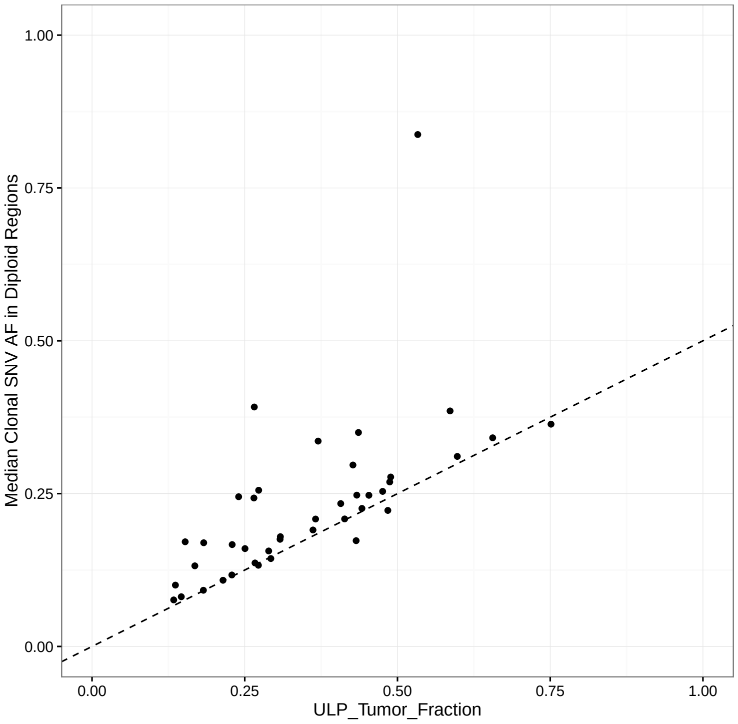

Supplementary Figure 16: Comparison between the cfDNA ULP-WGS tumor fraction and the median allele fraction of clonal SSNVs observed in diploid regions in matched cfDNA WES samples. The dashed line represents the expected relationship between tumor fraction and the allele fraction of clonal *heterozygous* SSNVs in diploid regions.

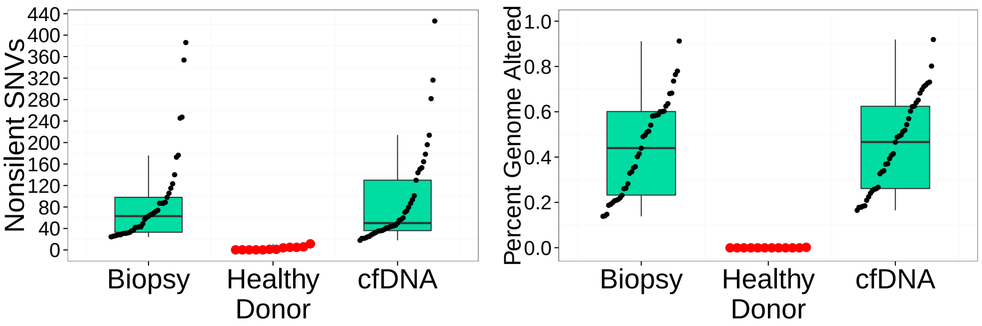

Supplementary Figure 17: Number of somatic SNVs (non-silent) and percent genome altered by SCNA in WES of cfDNA from cancer patients and healthy donors as well as WES of matched tumor biopsies. Boxplots indicate median and interquartile range (IQR, 25th and 75th percentiles); whiskers extends to 1.5*IQR. SSNVs were called by MuTect; percentage of genome altered was predicted by ABSOLUTE for cancer patients and TITAN for healthy donors.

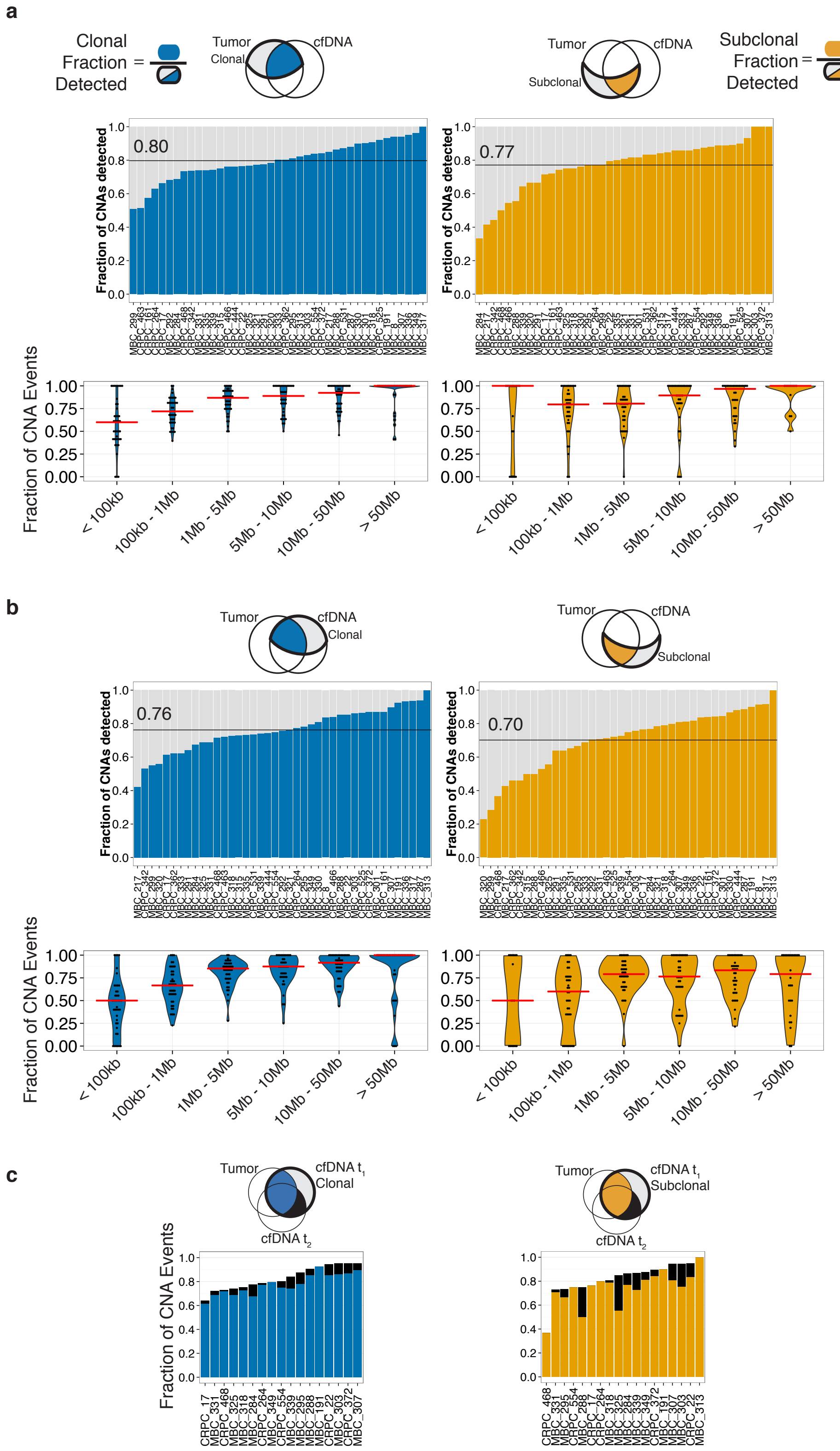

Supplementary Figure 18: Fraction of clonal and subclonal SCNA (a) detected in WES of tumor biopsies and confirmed in WES of cfDNA and (b) detected in WES of cfDNA and confirmed in WES of tumor biopsies. Clonal (0.9 CCF) and subclonal (< 0.9 CCF) events were separated into varying length scales: < 100kb, 100kb-1Mb, 1-5Mb, 5-10Mb, 10-50Mb, and >50Mb. (c) For 18 patients, SCNAs detected in cfDNA but not detected in the tumor were confirmed in a second blood draw (black). SCNAs were predicted using Absolute and determined as overlapping if 50% of the detected SCNA segment is confirmed as altered in the other sample.

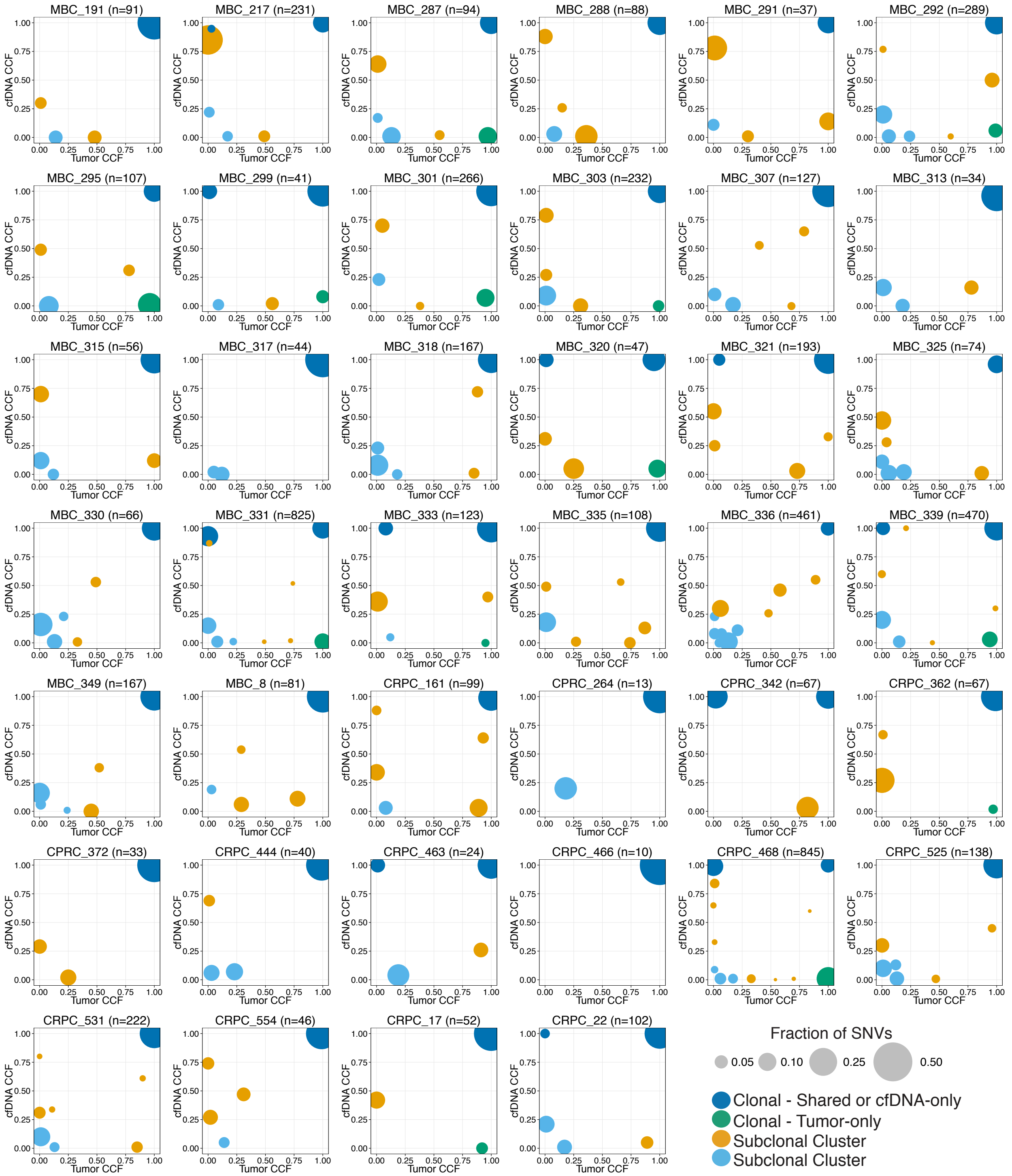

Supplementary Figure 19: Cancer cell fraction (CCF) for clusters of SSNVs detected in WES of cfDNA and matched tumor biopsies for 40 patients with metastatic breast cancer and patients with metastatic prostate cancer. Mutations were clustered by CCF for each pair of samples using a Phylogic. Error bars represent the 95% credible interval of the joint posterior density of the clusters. The number of mutations (excluding indels) are denoted in parentheses. Clonal (blue) SSNVs were defined as events having 0.9 CCF in both samples or 0.9 CCF in one sample but < 0.1 CCF in the other, otherwise, events were defined as subclonal (orange). Mutations having 90% power based on coverage in both samples are shown; clusters with < 2 mutations are excluded. Patient MBC_284 is shown in Figure 2.

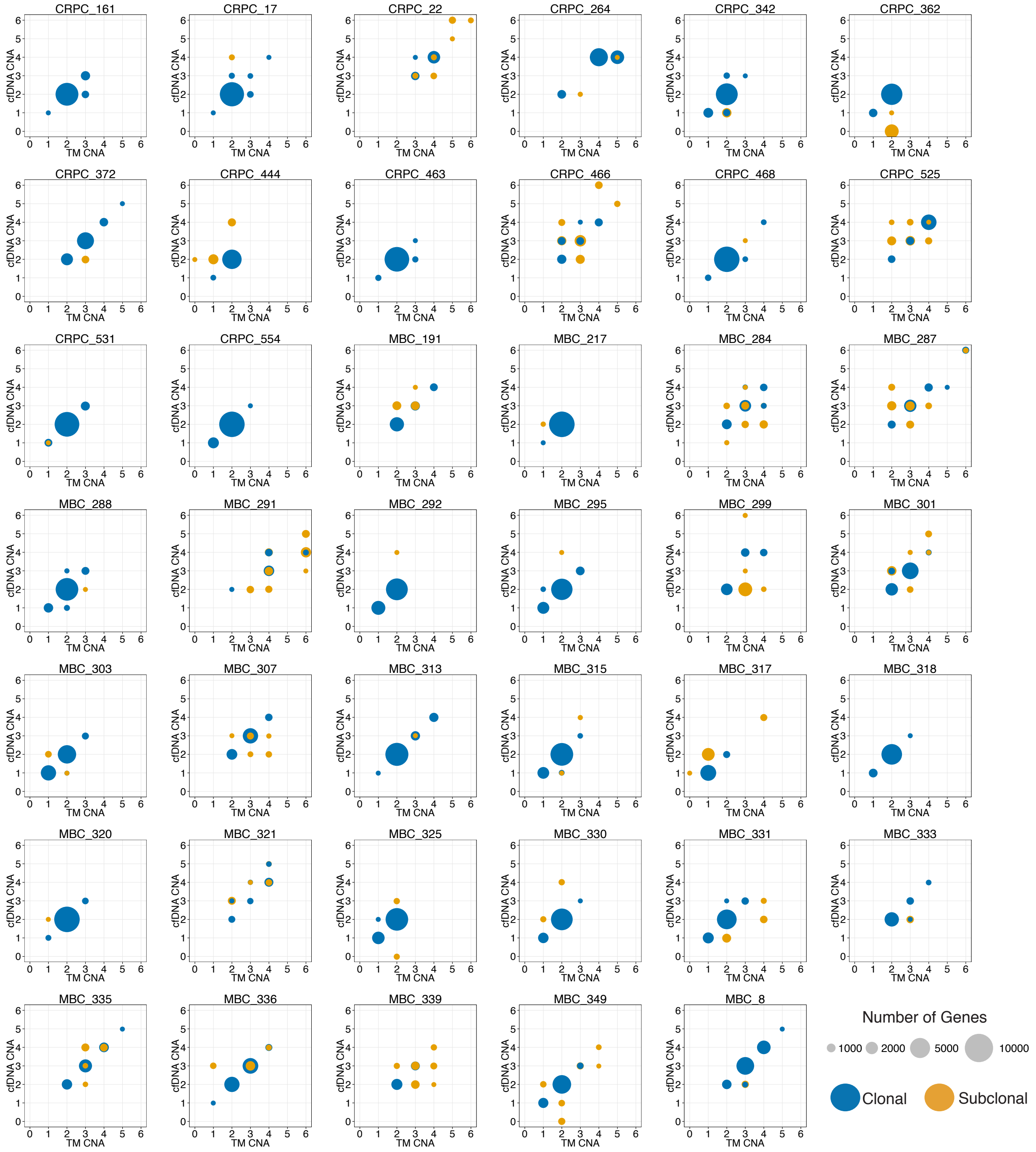

Supplementary Figure 20: Copy number alteration (CNA) comparison between WES of cfDNA and matched tumor metastatic biopsy for 41 patients. Clusters of genes altered by clonal (0.9 CCF) and subclonal (< 0.9 CCF) SCNAs, predicted by ABSOLUTE, from WES of cfDNA and matched tumor biopsies.

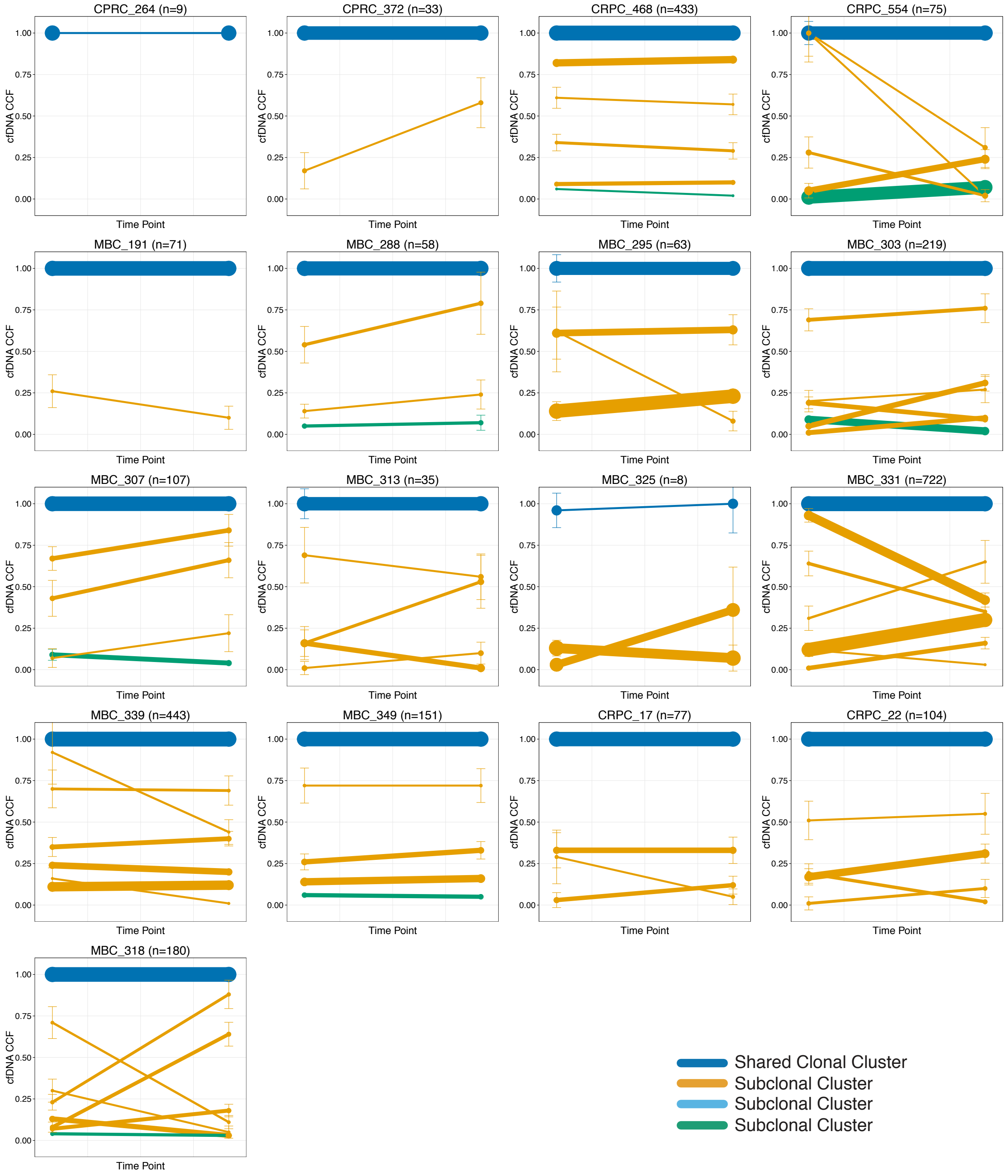

Supplementary Figure 21: Cancer cell fraction (CCF) for clusters of SSNVs identified in WES of cfDNA at time t_1 and t_2 for 17 patients. Mutations were clustered by CCF for each pair (t_1 and t_2) of samples using a Phylogic. Thickness of lines represent the relative number of SSNVs in the cluster. Patient MBC_284 is shown in Figure 2.

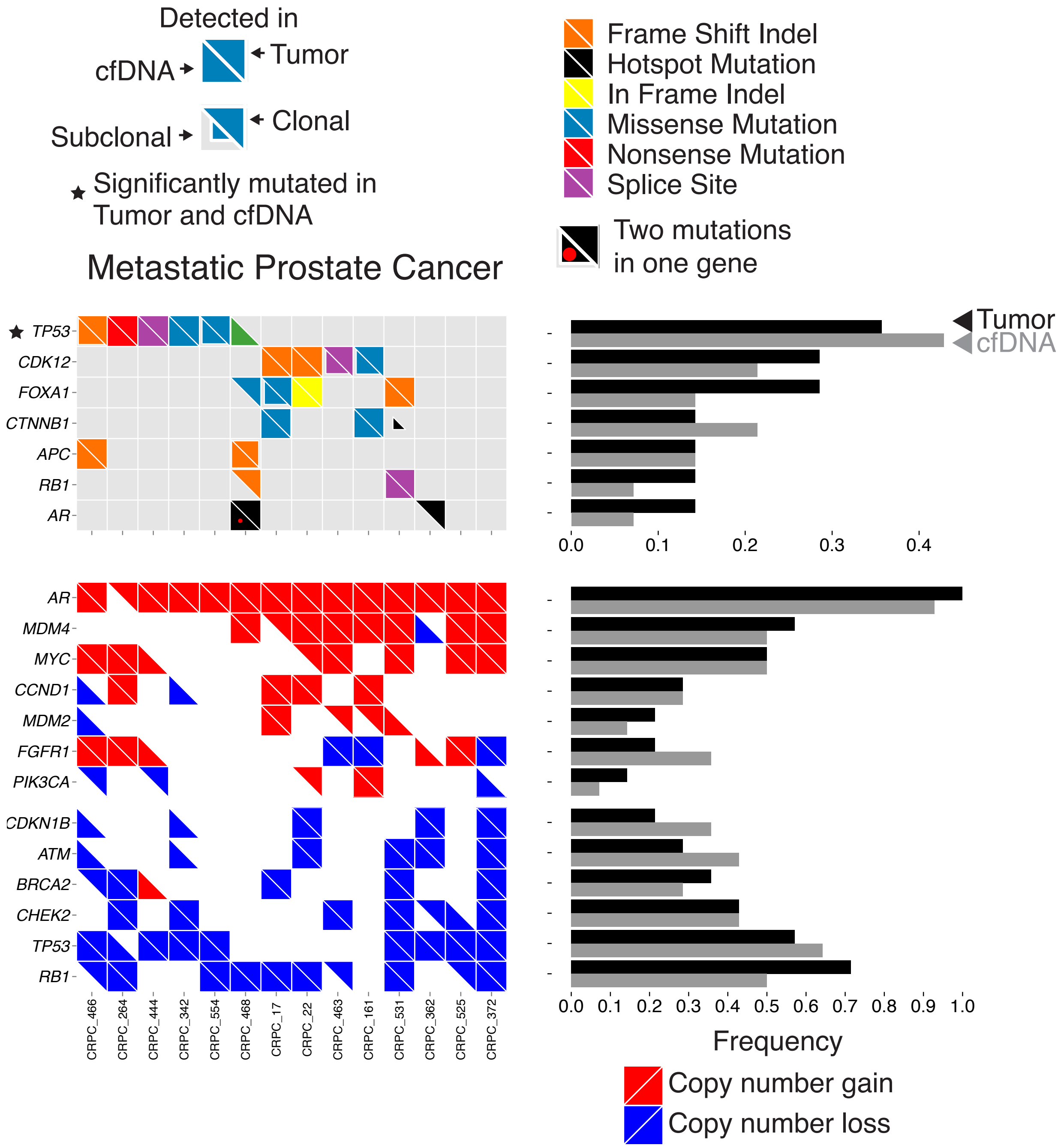

Supplementary Figure 22: The alteration status of significantly mutated genes predicted by MutSig2CV, focal SCNAs, and known cancer-associated genes are shown for cfDNA and tumor biopsies from 14 metastatic prostate (CRPC) cancer patients. Mutations were predicted using Mutect and SCNAs were predicted using ABSOLUTE. AR amplifications were predicted using TITAN/HMMcopy.

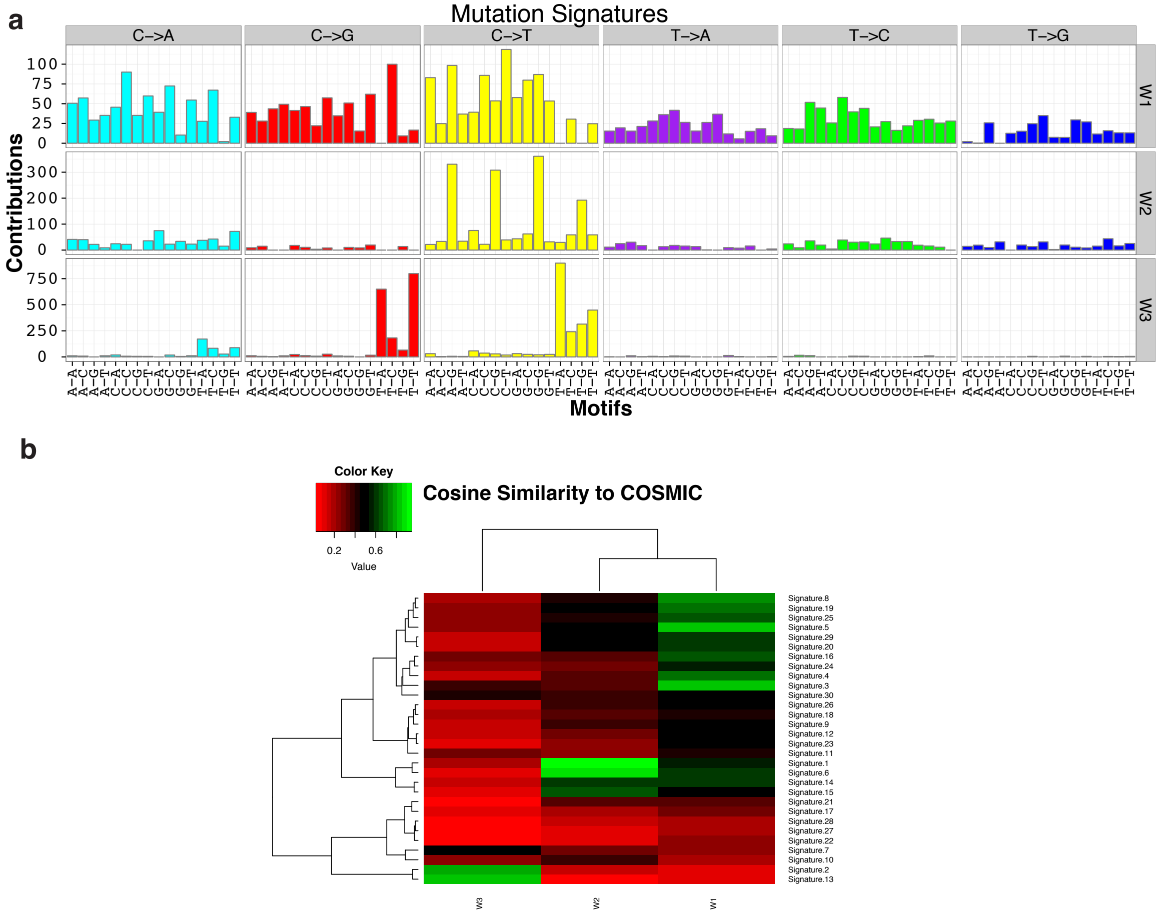

Supplementary Figure 23: Mutation signature analysis using a Bayesian non-negative matrix factorization approach. APOBEC (W3), BRCA-like (homologous recombination deficiency, W1), and aging (CpG, W2) signatures were predicted.

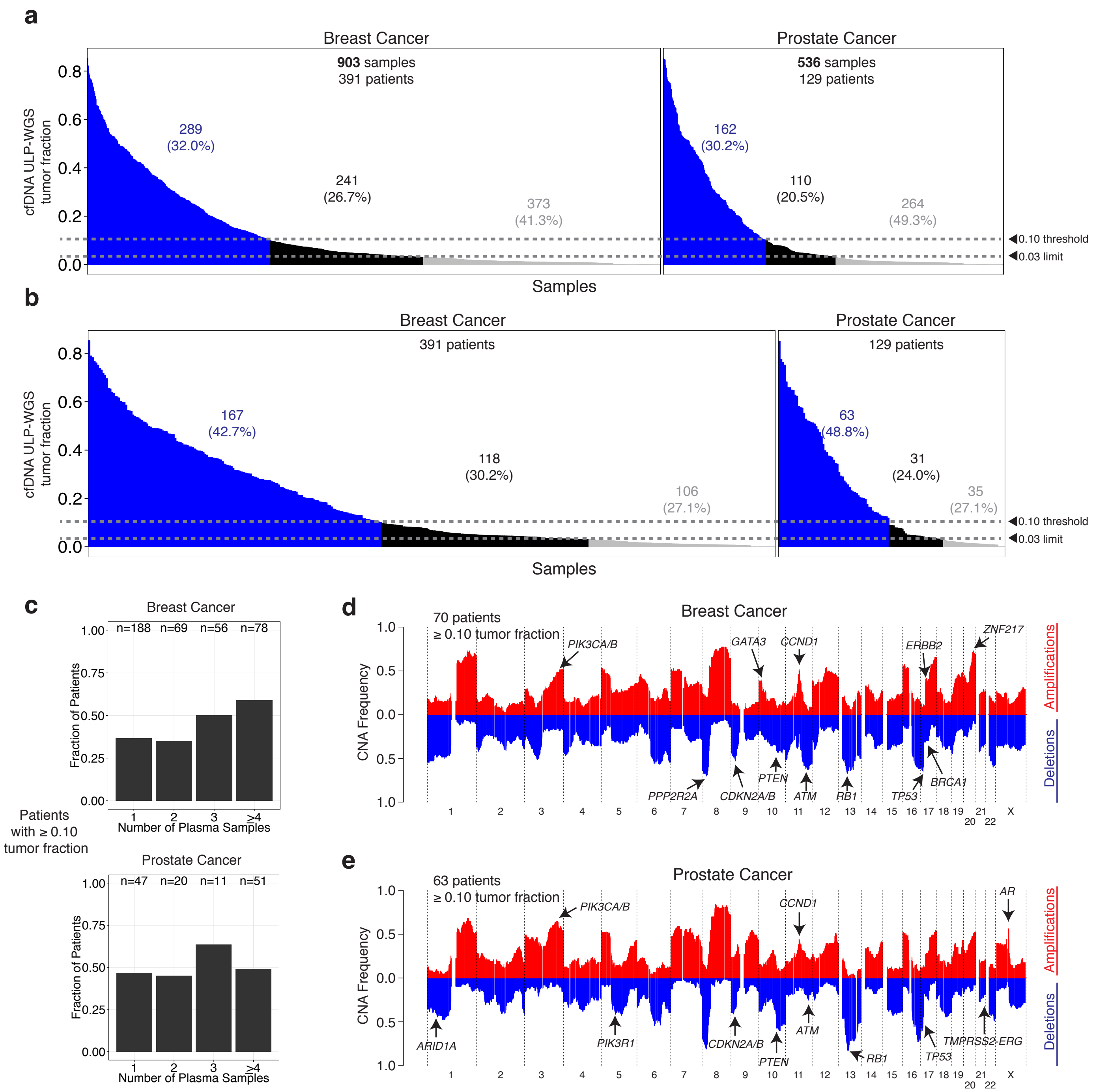

Supplementary Figure 24: Analysis of ULP-WGS tumor fraction and large-scale SCNAs. (a) ULP-WGS tumor fraction estimates for 1439 metastatic breast (MBC, 903) and prostate (CRPC, 536) cancer samples. Samples with ≥ 0.10 (blue), ≥ 0.03 and < 0.10 (black), and < 0.03 (grey) tumor fraction are indicated. (b) ULP-WGS tumor fraction estimates for samples the highest predicted tumor fraction and coverage ≥ 0.05 for each patient. (c) Fraction of patients having ≥ 0.1 tumor fraction, divided into groups containing 1, 2, 3, or ≥ 4 number of plasma samples collected. The total number of samples (n) within each of the three groups are indicated. (c-d) Gene-based frequencies of copy number alterations among patients with (d) metastatic breast and (e) prostate cancer predicted from ULP-WGS of cfDNA. Gene alterations were defined based on overlapping SCNA segments predicted from normalized read coverage at 1Mb bins. Only samples with ≥ 0.10 tumor fraction are included.

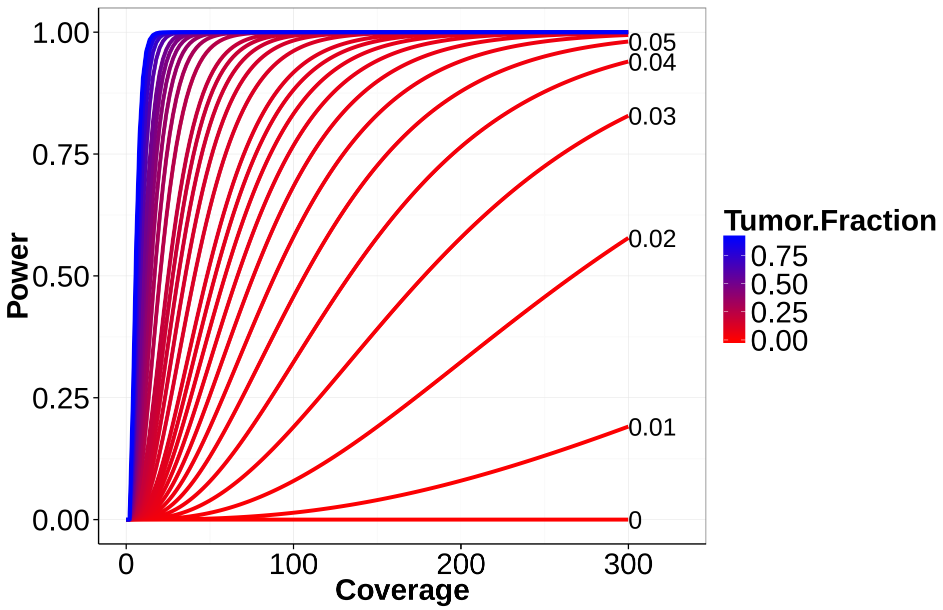

Supplementary Figure 25: Theoretical power to detect a mutation with tumor fraction based on coverage of whole exome sequencing. This analysis also applies to subclonal mutations with a specific cellular prevalence (i.e. cancer cell fraction). See Methods for power calculation.

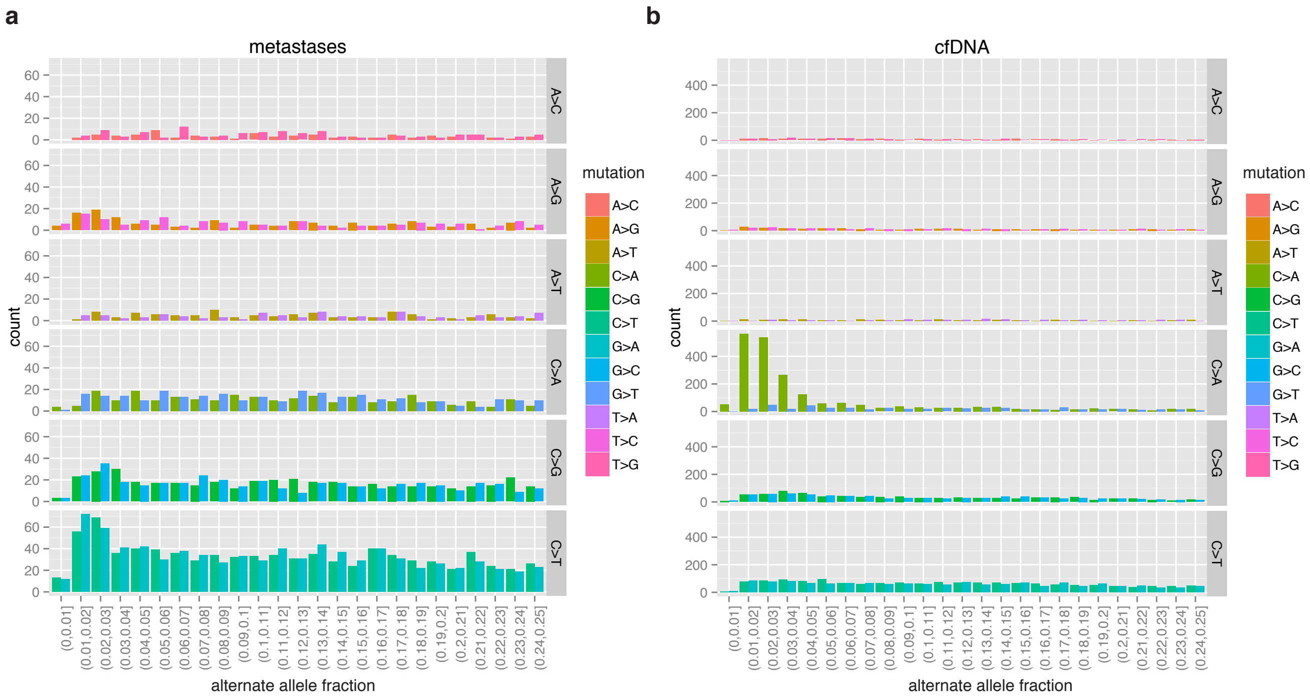

Supplementary Figure 26: Reference bias in cfDNA mutations before filtering. (a) Reference bias in tumor biopsies. Here, mutations are folded by strand (e.g. C>A represents both C>A and G>T mutations), and mutation frequencies are plotted as bars colored for non-folded substitutions (e.g. C>A and G>T SNVs are plotted as separate bars). Mutations are grouped in 1% allele fraction bins. Mutation frequencies are generally similar for the different reference bases within each folded context, indicating little reference bias as expected. (b) Reference bias in cfDNA before filtering. In cfDNA, for mutations in the C>A folded context, there are more C>A mutations than G>T mutations at low allele fractions, indicating reference bias, as previously described for cfDNA, and we applied additional filtering for C>A mutations at a reference C base.

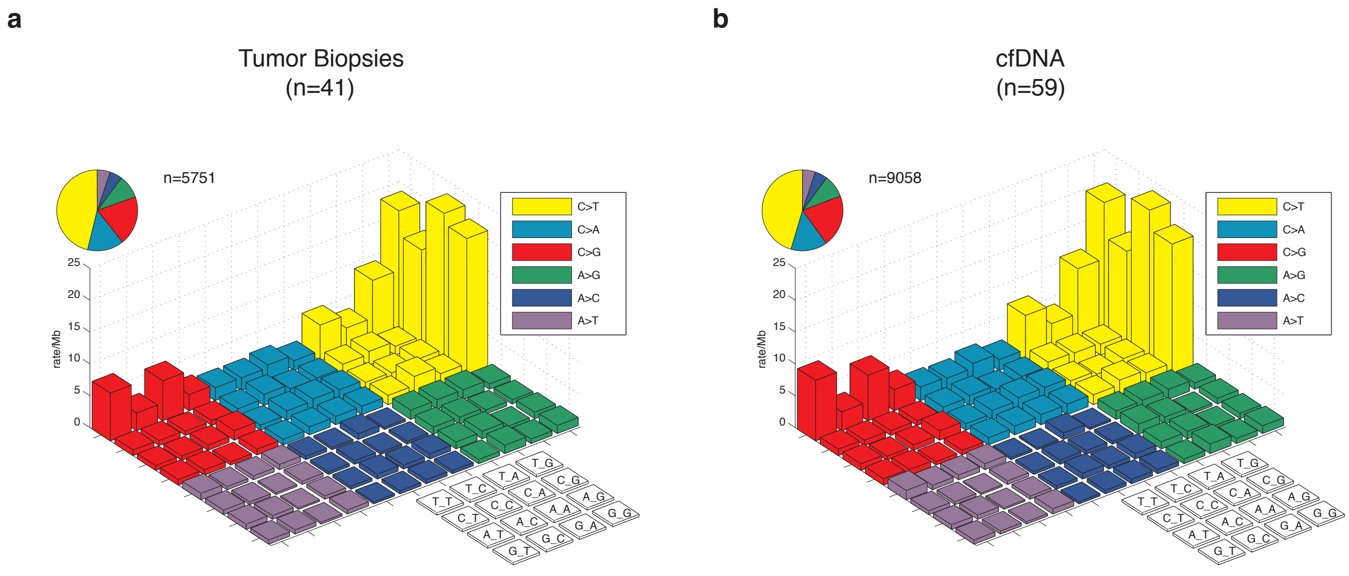

Supplementary Figure 27: Somatic mutation context in cfDNA and tumor biopsies. (a) Base contexts of mutations called in 41 tumor biopsies. Each color represents the base change (folded by strand, so mutations at reference G and T bases are reverse complemented), and each bar represents the frequency of a base change at a given mutational context with the indicated 5' and 3' bases. (b) Base contexts of mutations called in 59 cfDNA samples from 41 patients.
