# Supplementary material for: Scalable whole-exome sequencing of cell-free DNA reveals high concordance with metastatic tumors
Source: Nat Commun. 2017 Nov 6;8:1324. doi: 10.1038/s41467-017-00965-y (PMC5673918; doi:10.1038/s41467-017-00965-y)
Supplement: Supplementary file 2 — Description of Additional Supplementary Files [file 41467_2017_965_MOESM2_ESM.pdf]

Description of Additional Supplementary Files

File Name: Supplementary Data 1
Description: cfDNA metrics and ULP-WGS estimates for 1633 total samples from 1596 metastatic breast (974 samples, 391 patients) and prostate (622 samples, 129 patients) cancer patients and 27 healthy donors. sample_id: randomized breast (MBC) and prostate (CRPC) cancer patient IDs; identifiers ending with ".ctDNA" indicate the sample also has WES performed. Status:q indicates which samples are used for analysis - "Low Coverage"=coverage < 0.05 and excluded from analysis; "Below LOD"= < 0.03 tumor fraction; "Low Purity"= < 0.1 but ≥ 0.03 tumor fraction; "Pass"=≥ 0.1 tumor fraction. cancer_type: HD=healthy donor; CRPC=castration resistant prostate cancer; BRCA=metastatic breast cancer. subclone_fraction: proportion of tumor-derived DNA that a subclonal event is observed. fraction_genome_subclonal: fraction of the genome that is altered by subclonal events. fraction_cna_subclonal: proportion of predicted CNA events that are subclonal. mad: median absolute deviation of the copy ratio (not log2-transformed) differences between adjacent data points; indicator of data quality and coverage. er_status: for MBC samples, the clinical status of the estrogen receptor at the time of biopsy. ulp_wgs_coverage: the sequencing of ULP-WGS is computed as PF_READS_ALIGNED ∗ read_length ∗ 3 × 109; PF_READS_ALIGNED: pass-filter reads aligned. Tumor.Fraction: estimated amount of tumor fraction from ULP-WGS. Ploidy: estimated tumor ploidy from ULP-WGS. cfDNA_yield: total yield (ng) of cfDNA extracted from plasma. cfDNA_per_mL: yield (ng) of cfDNA extracted, normalized by each mL volume of plasma. Columns I through AF: Picard (version 1.1090) output from CollectAlignmentSummaryMetrics.

File Name: Supplementary Data 2
Description: Picard metrics for whole exome sequencing of cfDNA, tumor biopsies (TM), and germline blood normal (BN) from cancer patient (breast, MBC and prostate, CRPC) and healthy donors (HD). Metrics were generated using Picard (version 1.1090) CalculateHsMetrics. Samples from a second time point are indicated with "T2".

File Name: Supplementary Data 3
Description: ichorCNA benchmarking performance results of the in silico mixtures. a) "Merged" mixture experiment of 44 cancer patient cfDNA and 18 healthy donor samples mixed to generate 792 total mixtures. See Supplementary Figure 12 and Supplementary Methods for more details. b) "Exact" tumor fraction mixture experiment of 50 cancer patient cfDNA and deep whole genome sequencing of a healther donor (HD_2) mixed to generate 496 total mixtures. See Supplementary Figure 13 and Supplementary Methods for more details. Expected.Tumor.Fraction is computed using the predicted purity values from whole exome sequencing of the same cfDNA sample multiplied by the Expected.Mix.Fraction. Estimated.Tumor.Fraction and Estimated.Ploidy are estimated by ichorCNA for the mixture sample. Precision, Recall, and F1 are CNA performance metrics when compared to whole exome sequencing of the same cfDNA sample. Diff.Ploidy is true if the ploidy predicted by ichorCNA differs from the estimate from whole exome sequencing by > 0.75.

File Name: Supplementary Data 4
Description: Copy number alteration segment predictions for various sequencing data types for cfDNA and tumor biopies. (a) ichorCNA-predicted SCNA segments for ULP-WGS of cfDNA from 39 metastatic breast cancer (MBC) and 20 metastatic prostate cancer (CRPC) samples. num.mark is the number 1Mb bins in the segment; Seg.CN is the purity and ploidy corrected log 2 copy ratios; state.num is the predicted state given by the algorithm; state.name is the string representation of copy number (HETD=1 copy, NEUT=2, GAIN=3, AMP=4, HLAMP=5, HLAMP2=6, HLAMP3=>7 copies). subclone.status is the indicator of the segment being subclonal. (b) Titan3 allelic copy number segments for WGS (>10x coverage) of cfDNA from 7 samples. Allelic copy number is given in

columns: Copy_Number, MinorCN, MajorCN. (c) ichorCNA- predicted SCNA segments for WGS (1x coverage) of tumor biopsies from 22 MBC samples. (d) Titan3 allelic copy number segments for WES of 30 cfDNA and 23 tumor biopsy samples. (e) Absolute2 allelic copy number segments for WES of 59 cfDNA and 41 tumor biopsy samples. (f) ichorCNApredicted SCNA segments for ULP-WGS of cfDNA from 70 ER+ MBC patients. (g) ichorCNA-predicted SCNA segments for ULP-WGS of cfDNA from 63 CRPC patients.

File Name: Supplementary Data 5
Description: : Mutation annotation format (MAF) of mutations for WES of 59 cfDNA samples and 41 tumor biopsies from 41 metastatic breast and prostate cancer patients. (a) Mutect mutation calls were applied to each cfDNA and blood normal or tumor biopsy and blood normal pairs. Tumor_Seq_Allele2: mutant base t_alt_count and t_ref_count: number of alternate and reference alleles present in the tumor. (b) Strelka indel calls for WES of 59 cfDNA samples and 41 tumor biopsies. TAR: Reads strongly supporting1 non-indel allele for tiers 1 and 2 respectively. TIR: Reads strongly supporting1 indel allele for tiers 1 and 2 respectively (c) ABSOLUTE results for mutations from 41 patients with cfDNA (time 1) and tumor biopsy pairs and (d) 18 patients with WES of cfDNA time points t1 and t2. i_judgement: Initial MuTect judgement, KEEP if a mutation was initially called in a sample and REJECT if a mu- tant was not initially called but was evaluated due to force calling. alt,ref: alternate and reference allele counts. q_hat: estimated integer somatic copy number q at the site. HS_q_hat_1,HS_q_hat_2: estimated homologue-specific copy numbers at the mutant site. ccf_hat: estimated mutation cancer cell fraction (CCF). ccf_CI95_low,ccf_CI95_high: bounds for the 95% confidence interval of the mutation CCF. detection_power: power to observe 3 or more reads for a clonal mutation at multiplicity 1 (See Supplementary Methods) given the sample purity, local copy number and sample reference skew. purity: estimated sample purity. (e) Phylogic (2D) clustering results for WES of 40 patients with cfDNA (time 1) and tumor biopsy pairs and (f) a subset of 17 patients with cfDNA time points t1 and t2. (g) Phylogic (2D) clustering results for WES of MBC_284 considering the union of mutations called in the tumor biopsy as well as cfDNA time points t1 and t2. Results are presented for all possible pairs of samples. Note that in Phylogic table, the suffixes _1 and _2 denote the sample (e.g. alt_1 indicates the alternate allele count for sample 1), and the identities of sample 1 and 2 are indicated in the first two columns. CCF1, CCF2: clustered CCF of the mutation in sample 1 and sample 2 respectively.

File Name: Supplementary Data 6
Description: : Parameter estimates from ULP-WGS (ichorCNA) and WES (Absolute and Titan) of cfDNA and tumor biopsies from 41 metastatic breast (MBC) and prostate (CRPC) cancer patients. (a) Comparison of ichorCNA tumor fraction estimates with Absolute and Titan as shown in Fig. 1e and Supplementary Figure 15. WES.Weighted.CCF: the proportion of the subclonal event observed out of the total tumor-derived DNA (WES.Tumor.Fraction) in the sample, also called cancer cell fraction (CCF) in the bulk tumor context. The value provided is the weighted sum of the CCF based on the length of the subclonal segments. WES.Clonal.Cellular.Prevalence: the proportion of predicted clones out of the total tumorderived DNA (WES.Tumor.Fraction) in the sample. Prevalence of 1 is used for the clone that contains events that are present in the whole tumor-derived portion and is therefore not subclonal. Different.Ploidy was called TRUE if the difference between ULP.Ploidy and WES.Ploidy is > 0.75 or if is WES.Ploidy < 1.5. (b) Absolute parameters estimated for WES of cfDNA. Coverage.for.80.power: theoretical estimate of coverage required for 80% power to detect a mutation. (c) Titan parameters estimated for WES of cfDNA. CellularPrevalence: same definition as WES.Clonal.Cellular.Prevalence.

File Name: Supplementary Data 7
Description: Clinical information for 41 metastatic breast (MBC) and prostate (CRPC) cancer patients. Relative time (days) of plasma collection for cfDNA was computed with respect to the date of the

tumor biopsy collection. Biopsy_site indicates the anatomical site that the metastatic tumor was resected. For breast cancers, ER, PR, HER2 indicate the status of hormone receptors determined from the metastatic biopsies using immunohistochemistry.

File Name: Supplementary Data 8
Description: MutSig2CV results for (b,c) 27 metastatic breast (MBC) cancer and (d,e) 14 metastatic prostate (CRPC) cancer patients. (a) Cancer-associated gene lists, including SSNVs, amplifications, deletions for MBC and CRPC. codelen: the length of gene coding sequence. The number of mutations by category - nncd: non-coding, nsil: silent, nmis: missense, nstp: stop-codon, nind: indels, nnon: non-synonymous, npat: # of patients, nsite: # unique sites.
